# Core cooperative metabolism in low-complexity CO_2_-fixing anaerobic microbiota

**DOI:** 10.1093/ismejo/wraf017

**Published:** 2025-02-02

**Authors:** Guido Zampieri, Davide Santinello, Matteo Palù, Esteban Orellana, Paola Costantini, Lorenzo Favaro, Stefano Campanaro, Laura Treu

**Affiliations:** Department of Biology, University of Padova, Via U. Bassi 58/b, Padova 35131, Italy; Department of Biology, University of Padova, Via U. Bassi 58/b, Padova 35131, Italy; Department of Biology, University of Padova, Via U. Bassi 58/b, Padova 35131, Italy; Department of Biology, University of Padova, Via U. Bassi 58/b, Padova 35131, Italy; Department of Biology, University of Padova, Via U. Bassi 58/b, Padova 35131, Italy; Department of Agronomy, Food, Natural Resources, Animals and Environment (DAFNAE), University of Padova, Agripolis, Viale dell'Università 16, Legnaro, 35020, Italy; Department of Microbiology, Stellenbosch University, Private Bag X1, Matieland 7602, South Africa; Department of Biology, University of Padova, Via U. Bassi 58/b, Padova 35131, Italy; Department of Biology, University of Padova, Via U. Bassi 58/b, Padova 35131, Italy

**Keywords:** anaerobes, Co_2_ fixation, methane, cross-feeding, syntrophy, metagenomics, metatranscriptomics, metabolic modelling

## Abstract

Biological conversion of carbon dioxide into methane has a crucial role in global carbon cycling and is operated by a specialised set of anaerobic archaea. Although it is known that this conversion is strictly linked with cooperative bacterial activity, such as through syntrophic acetate oxidation, there is also a limited understanding on how this cooperation is regulated and metabolically realised. In this work, we investigate the activity in a microbial community evolved to efficiently convert carbon dioxide into methane and predominantly populated by *Methanothermobacter wolfeii*. Through multi-omics, biochemical analysis and constraint-based modelling, we identify a potential formate cross-feeding from an uncharacterised *Limnochordia* species to *M. wolfeii*, driven by the recently discovered reductive glycine pathway and upregulated when hydrogen and carbon dioxide are limited. The quantitative consistency of this metabolic exchange with experimental data is shown by metagenome-scale metabolic models integrating condition-specific metatranscriptomics, which also indicate a broader three-way interaction involving *M. wolfeii*, the *Limnochordia* species, and *Sphaerobacter thermophilus*. Under limited hydrogen and carbon dioxide, aspartate released by *M. wolfeii* is fermented by *Sphaerobacter thermophilus* into acetate, which in turn is convertible into formate by *Limnochordia*, possibly forming a cooperative loop sustaining hydrogenotrophic methanogenesis. These findings expand our knowledge on the modes of carbon dioxide reduction into methane within natural microbial communities and provide an example of cooperative plasticity surrounding this process.

## Introduction

In anoxic environments, carbon dioxide (CO_2_) can be converted into methane (CH_4_) by specialised archaea to produce energy. Hydrogenotrophic methanogenesis, an exclusive archaeal trait, uses oxidised carbon as the final electron acceptor for anaerobic respiration, generating energy and releasing CH_4_. It spontaneously occurs as a part of anaerobic degradation of organic matter in environments such as marshes, animal gut and the cow rumen, and it is also employed in biotechnological approaches for biogas production. Although this bioconversion has long been studied and characterised, it is now clear that its use and regulation in microbial consortia is largely governed by cooperative cross-species interactions. Understanding how these arise and which of them prevail at any condition is the crux for several issues such as tracking global carbon cycles [1], predicting climate trajectories [2], and achieving biological biogas upgrading for green energy production and storage [3].

In anoxic habitats, key microbial interactions are considered to take place in the form of cooperation. In its widest meaning, the term *syntrophy* denotes an obligately mutualistic metabolism [1, 4, 5]. A prominent example of anaerobic syntrophies entails the conversion of volatile fatty acids (VFA) to hydrogen (H_2_) and CO_2_ or formate by bacteria, used by archaea to fuel hydrogenotrophic methanogenesis [6–8]. Syntrophic acetate oxidising bacteria (SAOB) convert acetate to H_2_ and CO_2_, but thermodynamic constraints limit the energy yield, and continuous removal of the released H_2_ through methanogenesis is necessary to shift the equilibrium towards the simplest compounds, especially in high temperature and high ammonia conditions [8, 9]. Moreover, syntrophies may be necessary for coping with a wider range of biological or biochemical constraints, such as species-dependent biosynthetic capabilities [4, 5] and could also include facultative cooperation. However, how these interactions sustain CO_2_ fixation through hydrogenotrophic methanogenesis has not been yet elucidated. Indeed, in engineered microbial ecosystems characterised by high CO_2_-H_2_ content, SAOB co-exist with archaea despite thermodynamic unfavourable conditions for acetate degradation and are theoretically pushed towards homoacetogenic activity, which is favoured [10].

Much of current knowledge on microbial syntrophies derives from studies on cultivable representative strains or metagenomic analysis of natural and biotechnological systems. For a long time, the Wood–Ljungdahl (WL) pathway has been considered key to the establishment of bacteria-archaeal pairs mediated by acetate and CO_2_ transfer [11–13]. However, doubts have been recently raised on the metabolism of SAOB, as isolates have been proven to oxidise acetate in syntrophy with hydrogenotrophic methanogens yet lack crucial genes from the carbonyl branch of the WL pathway, namely the CODH/ACS complex (*acsABCDE* genes) [14]. Further, the same genes are frequently missing in metagenome-assembled genomes (MAGs) recovered from anaerobic digestion reactors [15]. A different pathway for syntrophic acetate oxidation has been previously proposed, denominated reductive glycine (RG) pathway [14], linking the methyl branch of the WL pathway with the glycine cleavage system (GCS). However, its role in syntrophic interactions is currently unclear.

Enrichment cultures represent a trade-off between synthetic mixed cultures and intricate natural communities in the study of microbial interactions. Unlike synthetic cultures, enrichment fosters the development of more controllable systems that closely mirror *in vivo* community assemblies. Moreover, as identifying crucial interactions a priori can be challenging, creating artificial consortia may lead to inaccuracies. Instead, selective enrichment, guided by nutrient provision, can promote the growth of metabolically interdependent species clusters, making it an attractive method for identifying inter-species relationships. This, combined with appropriate complementary tools, holds promise for uncovering the microbial interactions that shape natural ecosystems [16–19].

To pinpoint critical cooperative activity, the current study analysed an anaerobic community enriched on restrictive feeding to reduce the number of species. Cultures were grown with a synthetic medium with the aim of revealing the metabolic dependencies of individual microbes and to study the adaptation of the community to different carbon sources (acetate, H_2_/CO_2_). Transcriptomic data integrated into metagenome-scale metabolic models validated the potential interactions emerging from MAGs, successfully revealing bioconversion activity and growth requirements of the microbial consortium under investigation. Overall, the study demonstrated the strength of combining microbial community simplification with multi-omic analysis and modelling to unravel the complex metabolic interplay underpinning CO_2_ fixation into CH_4_.

## Materials and methods

### Microbial culture setup

A simplified community was obtained from a pilot-scale trickle bed reactor from the Technical University of Denmark, active in thermophilic biogas upgrading [20]. This reactor operated at 55°C on H_2_ and CO_2_ and spent digestate. The culture was grown in basal anaerobic (BA) medium and fed with pure CO_2_ and H_2_ in 1:4 volumetric ratio every 5 days, with the aim of adapting it to the feeding composition, enriching methanogenic and syntrophic species [21]. Six consecutive generations were carried out as described in Supplementary Document 1. To avoid abrupt transitioning from CO_2_ to acetate as carbon source, a final pre-adaptation was applied by providing both molecules in equimolar amounts over a 13-day period, using the same operational conditions applied in the final experiment (Supplementary Tables 1 and 3).

The experimental setup consisted of three distinct groups of triplicate fed-batch reactors undergoing a differential feeding regimen of acetic acid and CO_2_. Each reactor consisted of a 1 L glass bottle with a 350 ml working volume of BA medium supplemented with limited addition of yeast extract (0.2 g/L final concentration) and 20% v/v of inoculum from the simplified culture post-adaptation. The same molar quantity of carbon was provided to all reactors, however the ratio between the two carbon sources varied by group (Supplementary Table 3). Gas feeding was performed daily, whereas acetate addition was performed every 2 days according to the monitored consumption rates.

### Analytical measures

Microbial growth and biochemical activity were assessed over time by measuring OD at 600 nm, headspace gas production and composition, and VFA concentrations every 2 days following previously established methods [22]. OD was measured with a DTX 880 Multimode Detector plate reader (Beckman Coulter, Brea, CA, USA). Cell pellets from each reactor were sampled at the end of fed-batch operations, dried and weighted, to estimate biomass quantification. Gas composition profiles were obtained using a MicroGC (Agilent Technologies, Santa Clara, CA, USA) equipped with a thermal conductivity detector, a Molsieve 5 Å column, with argon used as a gas carrier, and a second PoraPLOT U column, with helium injection [23]. VFA measurements were performed on filtered samples (0.22-μm) with a Shimadzu Nexera HPLC (Shimadzu, Kyoto, Japan), equipped with a Phenomenex Rezex ROA-Organic Acid H^+^ column, set at 65°C. Acetaldehyde concentrations were measured with fluorometric assay (Sigma-Aldrich, St. Louis, MO, USA).

### Sample collection and sequencing

Samples were acquired after 7 and 9 days from reactor setting (point t1 and t2, respectively). Genomic DNA and RNA were co-extracted with RiboPure™ RNA Purification Kit (ThermoFisher Scientific, Waltham, MA, USA) following manufacturer’s instructions. Nucleic acid quality and quantity were assessed using Nanodrop2000 (ThermoFisher Scientific, Waltham, MA, USA) and Qubit Fluorometers (Invitrogen, Carlsbad, CA, USA). Co-extracted samples were split and processed independently: aliquots for RNA sequencing were treated using the RNA Clean & Concentrator-5 kit, including a DNase I treatment (Zymo Research, Irvine, CA, USA) for 15 min at room temperature.

Total RNA quality was evaluated using an Agilent 2100 Bioanalyzer with RNA 6000 Nano reagents and Chips (Agilent Technologies, Santa Clara, CA, USA). RNA Integrity Numbers (RIN) were ranging from 6.4 to 8.3. Ribosomal RNA was removed using FastSelect –5S/16S/23S Kit (QIAGEN GmbH, Hilden, Germany). Library preparation and sequencing was performed by the sequencing facility of the Department of Biology (Padova, Italy) using Nextera DNA Flex Library Prep Kit (Illumina Inc., San Diego, CA, USA) for DNA and TruSeq Stranded total RNA (Illumina Inc., San Diego, CA, USA) for RNA. Paired-end sequencing was performed on NovaSeq 6000 platform (Illumina Inc., San Diego, CA, USA). Genomic DNA libraries for Nanopore sequencing were done with the SQK-RBK004 rapid sequencing kit (Oxford Nanopore Technologies, Oxford, UK) and sequencing was performed with FLO-MIN106 R9 flow cell on a MinION device (Oxford Nanopore Technologies, Oxford, UK).

### Genome-centric metagenomics and metatranscriptomics

The short-read DNA sequences were filtered with Trimmomatic 0.39 [24] and BBTools [25], and next co-assembled with Megahit 1.2.9 [26]. Base calling for the long-read DNA sequences was performed with Guppy 5.0.11 (https://nanoporetech.com/) followed by co-assembly using Flye 2.9 [27] and error-corrected using the short-read sequences via Pilon 1.24 [28]. After that, the two assemblies were merged with quickmerge 0.3 [29] followed by further error correction with Pilon. At each step, we benchmarked assembly quality with Quast 5.0.2 [30]. To maximise the MAG completeness and quality, we adopted a multi-tool binning strategy, whereby we classified the contigs by CONCOCT 1.1.0 [31], MaxBin 2.2.7 [32], Metabat 2.15 [33], Metabat 2 2.15 [34], and Vamb 3.0.2 [35]. MAGs were de-replicated assessing their quality and calculating their coverage through CheckM 1.1.3 [36]. Taxonomic classification was estimated based on the Genome Taxonomy Database (GTDB) r207 by GTDB-Tk 2.1.0 [37]. Gene sequences were identified through Prodigal 2.6.3 [38] and evaluated with eggNOG-mapper 2.1.7 [39]. Average nucleotide identity (ANI) estimations were performed using dRep 3.4.0 [40].

Metatranscriptomics followed a previously used procedure [41] with modifications. Short RNA reads were filtered and aligned onto high-quality MAGs. RNA fragments were counted with HTSeq 2.0.2 [42] in stranded reverse mode (-a 0, --mode intersection-nonempty) against a combined GFF detailing contig and coordinates for the genes from all the high-quality MAGs. The resulting tables with RNA fragment counts per gene were then split by MAG and processed separately throughout all the subsequent steps in order to remove taxonomic composition variations from transcriptomic changes in individual taxa [43, 44]. The R package DESeq2 3.14 [45] was used for count processing and differential expression analysis. Transcriptome principal component analysis (PCA) was performed on the 500 most variable genes based on *rlog*-normalised counts. Differential gene expression between the H_2_/CO_2_ samples and acetate samples was performed with α=0.05 and lfcThreshold = 1, controlling for time point. For visualisation of gene expression, normalised fragments per kilobase (nFPK) were obtained by first normalising raw fragment counts by applying calculated size factors, then dividing by the corresponding gene length. In all the above procedures, sequence alignments were performed and manipulated by Bowtie 2.4.4 [46] and SAMtools 1.12 [47] with default parameters.

### Metagenome-scale metabolic modelling

Automatic genome-scale metabolic model (GEM) reconstruction was based on the proteomes of MAGs upon expanding gene annotation via KEMET [48]. Draft GEMs were built and gap-filled using gapseq 1.2 5a8e9859 [49], predicting biochemical reactions, transporters, and medium composition and ensuring growth in anoxic conditions. Non-default parameters were set as follows: -p all -b 200 in the gapseq find step; −u 200 -l 50 in the gapseq draft; −c “cpd00007:0” in the gapseq medium; −b 50 in the gapseq fill. When reconstructing the model for *Methanothermobacter wolfeii*, −t Archaea was also set in the gapseq find step and -b archaea was specified in the gapseq draft step. These parameters define a conservative level for inclusion of reactions in the draft GEM, avoiding incorrect introduction of pathways of uncertain presence.

Upon GEM refinement and application of experimental constraints (Supplementary Document 1), cooperative trade-off flux balance analysis (ctFBA) was used to estimate microbial growth rates and metabolic fluxes [50]. This approach approximates dynamic growth trajectories by identifying the shortest path between the inoculation point and the maximum growth regime, and was thus used to estimate metabolic activity in the growth phase [51]. Fluxes were calculated by minimising the total flux sum through the exchange reactions, thus imposing a parsimonious usage of environmental resources. This was realised through the calculation of a minimal medium satisfying the growth regime identified by ctFBA. CoCo was used to integrate metatranscriptomics count data, normalised and transformed on a logarithmic scale through DESeq2, into individual community GEMs [41]. The parameter α was set upon grid search over the range [0.1, 10], whereas α was set to 1.0. Analogously, the community trade-off was explored over the values {0.1, 0.2, …, 1.0}. Community-level growth rates, obtained by semi-logarithmic interpolation of OD values, were used as a reference during parameter selection based on Pearson’s correlation with model-predicted community growth rates. All the simulations were performed in Python 3.6 with the CPLEX 12.8 solver (https://cplex.org), rounding to 0 all resulting flux values lower than the numerical tolerance [10-6].

## Results

### CO_2_-fixing community simplification

To identify essential cooperative interactions between methanogenic archaea and cooperating bacteria, we set out to create a simplified microcosm of a real CO_2_-fixing ecosystem. CO_2_ bioconversion takes place during hydrogenotrophic methanogenesis, with mutual cooperation of multiple taxa. Here, we aimed at obtaining a reduced set of microorganisms comprising hydrogenotrophs and co-existing bacteria, providing an *in vivo* model of syntrophic methanogenic consortia.

**Figure 1:**
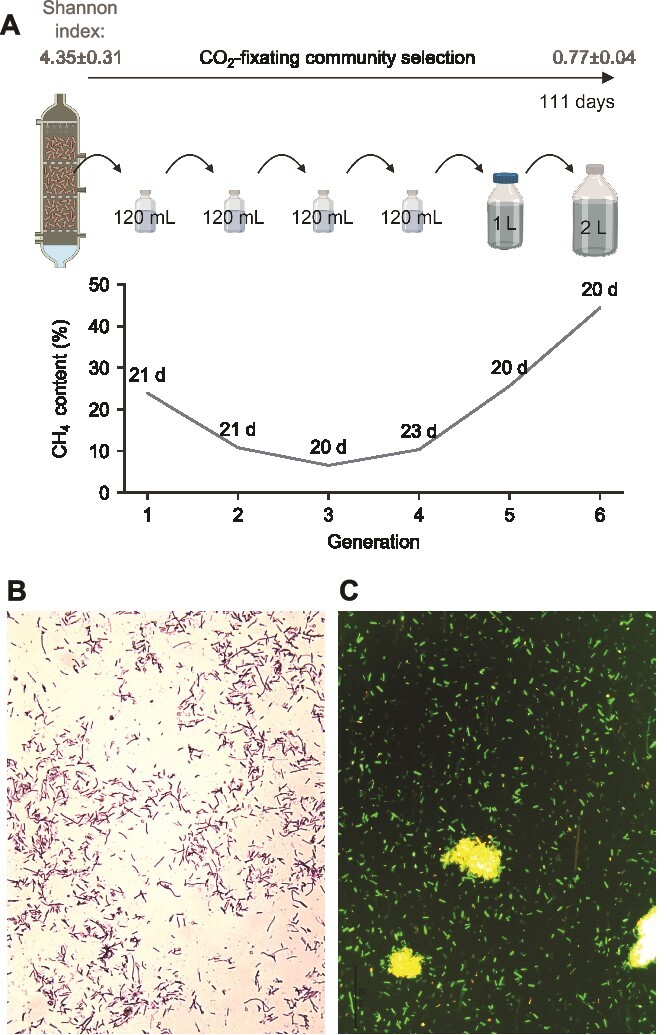
CO_2_-fixing community selection. (A) Microbial enrichment workflow illustrating the percentage of CH_4_ produced in each generation and the number of days to reach such production once reinoculated from the previous generation. CH_4_ production was monitored even after reinoculation into the next generation. On the top, initial and final community α-diversity expressed as mean and standard deviation of the Shannon index. (B) Final microbial morphology shown by gram staining. (C) Final microbial viability obtained by fluorescein diacetate and propidium iodide (FDA-PI) double staining assay.

Focussing on the selection of a CO_2_-fixing microcosm, we cultured a consortium originating from a thermophilic trickle-bed reactor over six generations of re-inocula by providing gaseous H_2_ and CO_2_ as the sole compounds exploitable for energy production (Fig. 1A, Supplementary Table 1). By monitoring microbial growth and CH_4_ production over each generation, we ensured that methanogens got selected together with potential partners necessary to their survival (Fig. 1A). To minimise the chance of selecting competitors, a minimal medium was used (Methods). This process extended for a total of 111 days, leading to an almost six-fold reduction in α-diversity from the originally sampled trickle-bed reactor to the end of the last generation (Shannon index from 4.35 ± 0.31 to 0.77 ± 0.04). 16S rRNA gene amplicon sequencing indicated the dominant abundance of a *Methanothermobacter* species in the consortium along with several bacteria during the fourth generation (Supplementary Table 2). At the conclusion of the selection, staining and viability assays verified the presence of widespread Gram-positive rod-shaped microbes (Fig. 1B-C). These characteristics delineated a profile compatible with that of *M. wolfeii* [52–54]. This methanogen is commonly found in thermophilic anaerobic digesters and biogas upgrading systems utilising the hydrogenotrophic pathway, with some strains also capable of using formate for energy production [55].

### *M. wolfeii* dominates a diverse community

**Figure 2:**
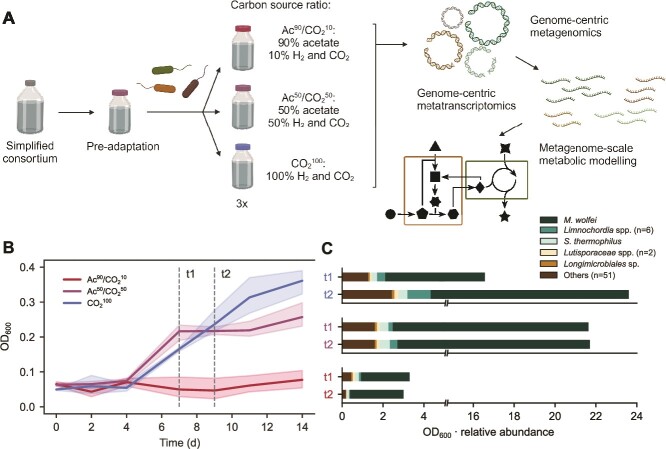
Microbial population response to alternative carbon source ratios. (A) the simplified community was fed with a minimal medium whose carbon source was either predominantly acetate, mixed acetate and CO_2_, or only CO_2_. The established communities were characterised through DNA and RNA shotgun sequencing, followed by metagenome-scale metabolic model development. (B) Growth dynamics of the microbial communities. Vertical dashed lines indicate the sampling points t1 and t2. (C) Estimates of absolute microbial abundances calculated by multiplying OD and relative abundances obtained from DNA shotgun sequencing.

Having established a simplified methanogenic ecosystem, its assembly mechanisms were characterised by testing the responses to carbon source availability. A fed-batch experiment was designed (Fig. 2A) where alternative substrates were defined according to carbon source ratios: (i) ~90% acetate and 10% CO_2_ mixture (Ac^90^/CO_2_^10^); (ii) 50% acetate and 50% CO_2_ (Ac^50^/CO_2_^50^); and (iii) 100% CO_2_ (CO_2_^100^) (Supplementary Table 3). The substrate volumes were set so as to provide the same C-equivalent moles in all the conditions. Hydrogen was fed in a 1:4 ratio with CO_2_. Following a preliminary adaptation with mixed feeding (Methods and Supplementary Fig. 1), triplicate biological samples were collected during the growth phase at two distinct time points (here indicated as t1 and t2) for each condition to track microbial community composition and activity (Fig. 2B). As metabolic cross-feeding in the considered syntrophic interactions is hypothesised to be bidirectional depending on the external conditions [10], such a design served two main goals. On the one hand, high H_2_ and CO_2_ availability is supposed to lead to the emergence of bacteria providing *M. wolfeii* with essential nutrients, overcoming potential archaeal auxotrophies through a mutually beneficial exchange. In contrast, in CO_2_-scarce conditions bacteria capable of acetate-to-CO_2_ conversion should emerge, if present, providing a basis for hydrogenotrophic methanogenesis. In the latter case, a mutually beneficial exchange could arise as a consequence of bacterial auxotrophies (e.g. for some specific organic compounds or amino acids). Final optical density (OD) was proportional to H_2_ and CO_2_ amounts across conditions and CH_4_ production was found to be significantly lower in Ac^90^/CO_2_^10^ (Supplementary Table 3).

The composition of the established consortia was revealed by metagenomic co-assembly and binning integrating short- and long-read sequencing. A total of 61 taxa were obtained, comprising 47 high-quality and 14 medium-quality MAGs, in accordance with MIMAG standards [56] (Supplementary Data 1). As anticipated, the dominant microbe was taxonomically assigned to *M. wolfeii* and exhibits relative abundances ranging from 72.0 to 88.6%. The MAG is constituted by a single contig spanning 1.67 Mbp, with a predicted completeness of 99.7% and no contamination (Supplementary Data 1). Although the overall microbial growth and CH_4_ production were minimal in the acetate-rich medium, consistent with the hydrogenotrophic nature of *M. wolfeii*, its relative abundance remained high, suggesting that it influenced community dynamics even in this extreme feeding condition (Fig. 2C).

Despite the highly selective cultivation applied, numerous bacterial species were identified to constitute a functionally active low-abundance segment of the community. The second most abundant MAG, classified as *Sphaerobacter thermophilus*, accounted for 1.6–5.7% of the community. The species was originally isolated from sewage sludge and is cultivated on protein-rich media at thermophilic conditions [57]. Its presence in the current study suggests the identification of a facultative anaerobic strain, speculated also for other *Sphaerobacter* species [58]. Among other taxa contributing to the community composition, six MAGs belong to the *Limnochordia* class, collectively accounting for an average of 2.1% abundance. The taxon harbours primary candidates for syntrophic interactions with hydrogenotrophic archaea, in particular *Limnochordia* sp. 5, which was assigned to the GTDB species DTU010 sp002391385. As previously indicated by statistical association, imaging, and computational modelling, such a species has been predicted to play a primary role in sustaining a minimal CO_2_-converting consortium through metabolic exchanges [10]. Further, this MAG scored an ANI of 99.5% with a *Clostridia* previously identified as an acetate utilizer via stable isotope probing [59].

Considering that microbial syntrophs can cooperate while coexisting in abundances differing even of 10 folds, the established community composition was thus relevant to capture potential cooperative behaviour between *M. wolfeii* and some of the bacterial species [60, 61]. In Ac^90^/CO_2_^10^, the lack of a clear community growth phase could be explained by slow dynamics resulting from the long adaptation period coupled with the drastic carbon source change [62]. Indeed, computationally estimated microbial growth rates for several taxa positively correlated with the amount of acetate provided (Supplementary Fig. 6). Thus, given the detected methanogenic activity, we scrutinised more in depth the underlying archaeal metabolism.

### *M. wolfeii* regulates methanogenesis towards syntrophy in CO_2_ scarcity

**Figure 3:**
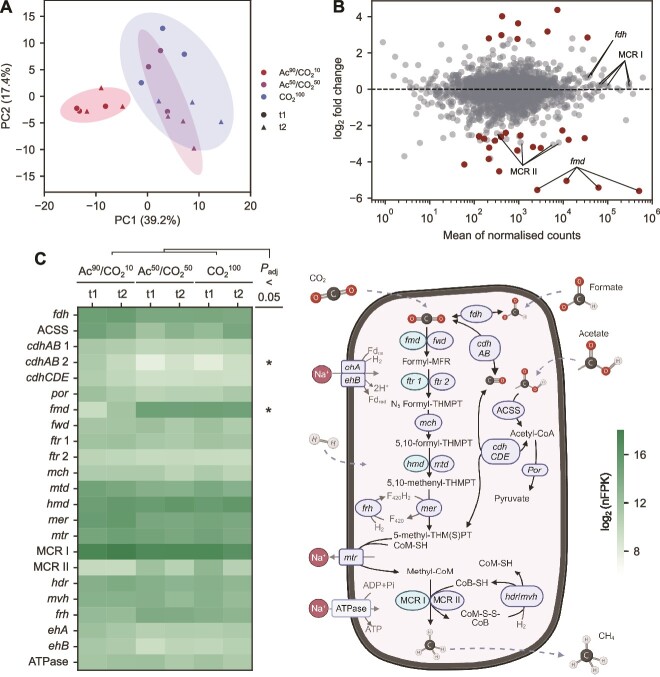
*M. Wolfeii* response to alternative carbon source ratios. (A) Principal component representation of *M. Wolfeii* transcriptomic activity across conditions. (B) Comparison between gene expression in Ac^90^/CO_2_^10^ and CO_2_^100^, with differentially expressed genes marked in full opacity. (C) Mean gene expression involved in carbon assimilation and methanogenesis across conditions. Asterisks mark enzymes with at least one differentially expressed gene with adjusted *P* value < 0.05. The identified genes are illustrated in the diagram on the right. Abbreviations: Methanofuran (MFR), tetrahydromethanopterin (THMPT), (THM(S)PT), coenzyme M (CoM), sulfhydryl coenzyme M (CoM-SH), sulfhydryl coenzyme B (CoB-SH), heterodisulfide (CoM-S-S-CoB).

To understand the strategies utilised by *M. wolfeii* to cope with different levels of CO_2_, we examined its functional activity by genome-centric metatranscriptomics. In keeping with the metagenomics results, it accounted for the majority of the transcriptional activity in all the nutritional conditions (Supplementary Table 3). Low-dimensional transcriptome representation revealed a large overlap of Ac^50^/CO_2_^50^ and CO_2_^100^ samples, with a net separation with respect to Ac^90^/CO_2_^10^ (Fig. 3A). Thus, the increased availability of the gases did not significantly shift the activity of *M. wolfeii* as did the change in major carbon source to acetate, as expected. The similar response to pure CO_2_ and mixed feeding could in principle suggest that the carbon source provided as acetate could be partially converted to CO_2_ by SAOB and made available to the methanogen, but the low bacterial abundance makes this effect likely marginal. Moreover, in both these conditions a separation by time point can be observed (Fig. 3A), reflecting a globally higher expression in t1 both over the methanogenesis pathway and key genes involved in the translation process such as the large subunit ribosomal protein L13 (*rpl13*), translation initiation factor IF-2 (*infB*), and translation initiation factor 2 subunit 1 (*eif2a*) genes (Supplementary Data 2). Such a separation might be ascribed to the medium depletion occurring at late stage growth, particularly for CO_2_^100^ that shows the highest cellular density and the most evident separation between time points.

To determine in detail the consequences of carbon source availability for energy generation, gene annotation was manually curated and relevant duplicated genes (homologs) were identified. Differential expression analysis between Ac^90^/CO_2_^10^ and CO_2_^100^ revealed that most genes of the hydrogenotrophic methanogenesis pathway were not significantly affected by the shift in carbon source, with few exceptions (Fig. 3B). The pathway starts with formyl-methanofuran (formyl-MFR) as the first intermediate produced by the formyl-methanofuran dehydrogenase (*fmd/fwd*) genes and culminates with the release of CH_4_ by coenzyme-M reductase (MCR I and II), which is the key rate-limiting step (Fig. 3C, Supplementary Document 1). In Ac^90^/CO_2_^10^, *fmd* expression decreased nearly 45-fold (*fmdB,C,E*, adjusted *P* < .01), whereas MCR II decreased nearly seven-fold (*mrtB,D,G,A*, *P* value <0.05) compared to CO_2_^100^. In contrast, genes for carbon assimilation were upregulated in Ac^90^/CO_2_^10^. Genes coding for carbon monoxide dehydrogenase (CODH) were upregulated, in particular one cluster of *cdhAB* genes increased around eight times and the difference was selectively statistically significant (*cdhA,B*, adjusted *P* < .01).

Isoenzymes of methyl coenzyme-M reductase displayed a marked differential activity, with MCR I having over 100-fold as many nFPK as compared to MCR II in all the conditions (Fig. 3B). This difference between homologs was higher when cultures were fed with acetate and decreased with increasing amounts of CO_2_. As evidenced in previous studies, preferential usage of MCR I is a marker of syntrophic growth with H_2_-releasing bacteria where the archaeon accepts alternative electron sources made available by the partners [55, 63]. Moreover, the high expression level of formate dehydrogenase (*fdh*) and its moderate upregulation (log_2_-fold change = 0.7) in Ac^90^/CO_2_^10^ although non-significant suggest that, in CO_2_-limited conditions, *M. wolfeii* could exploit the formate released by syntrophic bacteria for growth. This finding is of particular interest because, together with MCR I expression, it suggests an alternative strategy for survival of this archaeal strain, previously documented in other isolated *Methanothermobacter* species [64]. Together, these patterns point to syntrophic exchanges with one or more bacterial partners.

### One *Limnochordia* species emerges as a key syntroph

**Figure 4:**
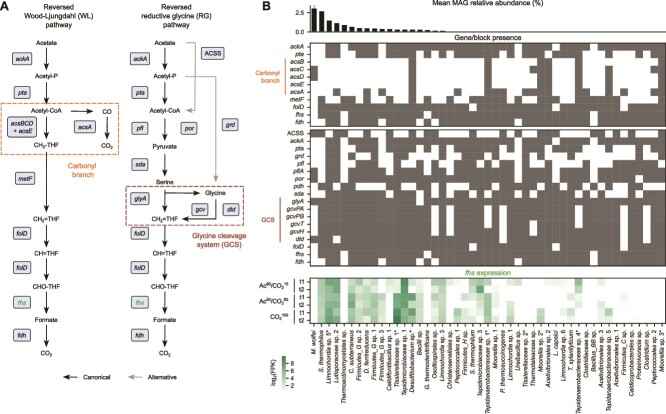
WL and RG pathway completeness and activity across taxa. (A) Graphical illustration of the bacterial WL and RG pathways. (B) WL and RG genes identified in high-quality MAGs, ordered by decreasing mean MAG abundance. On the bottom, *fhs* expression across MAGs and conditions. MAGs having either the WL or RG pathway complete are marked with an asterisk.

To identify potential partners of *M. wolfeii*, we first took into consideration the genomic completeness of the canonical and alternative WL pathway. In both pathways, formate is the last intermediate and could thus replace CO_2_ in syntrophic exchange. Overall, only few bacterial MAGs encoded a complete WL pathway, as frequently the carbonyl branch was completely missing and all of them had mean relative abundance below 1% in all the conditions (Fig. 4). Although this absence would prevent homoacetogenic function, the lack of these genes is not a strict indication against their putative SAOB role [14]. Rather, this is an indication that many bacteria in this community might employ enzymes not associated with the canonical WL pathway, as per the proposed RG pathway. Indeed, RG genes were widespread (Fig. 4) and the GCS showed a high degree of completeness across bacterial MAGs, likely also justified by its connection with amino acid metabolism. AMP-forming Acetyl-CoA synthetase (ACSS) was present in most MAGs lacking the phosphate acetyltransferase (*pta*)—acetate kinase (*ackA*) module, where it could account for the generation of acetyl-CoA from acetate. Among the most abundant bacterial MAGs, *S. thermophilus* encodes ACSS and pyruvate dehydrogenase to produce pyruvate but lacks L-serine dehydratase (*sda*) to fulfil the complete RG pathway. *Limnochordia* sp. 5 harbours most of the relevant genes. While lacking the *acs* genes encoding the CODH/ACS complex, it can produce all the alternative enzymes for interconverting acetyl-CoA and pyruvate and all genes of the RG pathway. Genes for different steps of the RG pathway were present in close locations in this MAG, with *ack*A (contig44_230), formate--tetrahydrofolate ligase (*fhs,* contig44_259*)*, methylenetetrahydrofolate dehydrogenase (NADP+) / methenyltetrahydrofolate cyclohydrolase (*folD*, contig44_269), and dihydrolipoyl dehydrogenase (*dld,* contig44_260) located in the same contiguous region spanning ~40 kbp (Supplementary Fig. 3).

Beyond functional potential, by using RNA-seq data we inspected the expression of the *fhs* gene, encoding formate-tetrahydrofolate ligase (FTHFS), whose expression can function as a marker for determining the SAO activity (Supplementary Document 1) [65]. Here, we considered non-normalised FPK values to capture the contribution of both abundance of the MAG and expression of the gene to the total enzyme activity. *Limnochordia* sp. 5 displayed a high *fhs* expression level both in CO_2_^100^ and Ac^90^/CO_2_^10^, along with other low-abundant bacteria (Fig. 4B). Furthermore, computationally estimated replication rates for this species increased in Ac^90^/CO_2_^10^ (Supplementary Fig. 6), consistent with the hypothesis that acetate represents a resource and with the high sequence similarity of a syntrophic acetate oxidiser [59]. Altogether, genomic and transcriptomic evidence therefore supports *Limnochordia* sp. 5 as a primary syntroph in the established consortium, motivating more detailed analysis of its functional activity.

### Activity of the putative RG pathway in *Limnochordia* sp. 5

**Figure 5:**
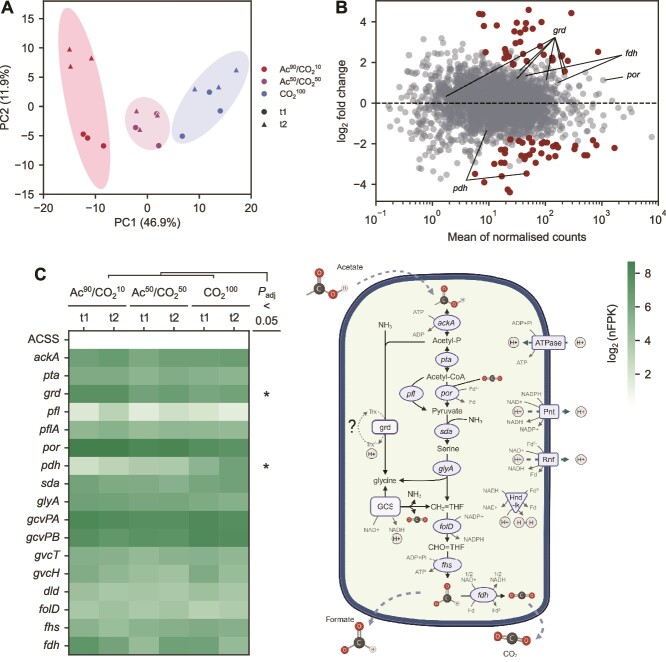
Acetate oxidation and homoacetogenic activity of *Limnochordia* sp. 5 under alternative carbon source ratios. (A) Principal component representation of *M. Wolfeii* transcriptomic activity across conditions. (B) Comparison between gene expression in Ac^90^/CO_2_^10^ and CO_2_^100^, with differentially expressed genes marked in full opacity. (C) Mean gene expression of genes for the RG pathway. Asterisks mark enzymes with at least one differentially expressed gene with adjusted *P* value < 0.05. The identified genes are illustrated in the diagram on the right, illustrating the genes and reactions identified or absent in the MAG. Abbreviations: Hnd-like hydrogenase (Hnd-lk), tetrahydrofolate (THF).

Based on the results of the WL and RG pathway analysis, we analysed gene expression in *Limnochordia* sp. 5 which globally adjusts to varying carbon source ratios (Fig. 5A). The high variance explained by PC1 and the group relative position suggest that this species considerably shifts its activity in response to the different carbon sources. Though some differences between timepoints can be seen, expression of the RG pathway was consistently maintained across all conditions, with most genes changing less than two-fold in Ac^90^/CO_2_^10^ compared to CO_2_^100^ (Fig. 5B). This contrasts with the marked separation of the transcriptome in the principal component space. Given the stable expression of the pathway with both feedstocks, its central role in carbon metabolism and the higher abundance of this MAG in the CO_2_^100^ condition, it is plausible that it can act in both directions, akin to the canonical WL, and might therefore allow survival of the organism on CO_2_ only. Under this hypothesis, *Limnochordia* sp. 5 may exert both acetate oxidation and homoacetogenic activity, although the energy balance of the process cannot be reliably performed based only on metagenomic data and no isolate available for biochemical assays.

Differentially expressed genes in the RG pathway further indicate a bidirectional usage consistent with the prevalent carbon source (Fig. 5B). Genes encoding components of the glycine reductase complex increased more than two-fold in Ac^90^/CO_2_^10^ in comparison to the other conditions (*grdE*, adjusted *P* value <0.05, *grdA,B, P* value <0.05). This complex could convert acetyl phosphate to glycine allowing acetate oxidation through the RG pathway. The direction of this reaction remains to be clarified due to the required reduction of a thioredoxin. Moreover, enzymes interconverting pyruvate and acetyl-CoA demonstrated opposite trends, possibly owing to a specific directionality. A pyruvate-ferredoxin/flavodoxin oxidoreductase coding gene (*por*, contig38_1128) was stably among the highest expressed genes in the organism. Pyruvate-ferredoxin oxidoreductase has been documented to be a viable pyruvate synthase in *Moorella thermoacetica* [66]. A role in the oxidative WL pathway would require a counterintuitive investment of reduced ferredoxin. It is alternatively possible that pyruvate formate-lyase could be used to generate pyruvate [14], although its expression was notably lower and non-significantly increased with acetate feeding. On the contrary, pyruvate dehydrogenase decreased four-fold with acetate feeding (*pdhA*, adjusted *P* value <0.01) and it could represent a mechanism for generation of acetyl-CoA in the alternative WL pathway for acetogenesis in the CO_2_-rich condition, perhaps supporting pyruvate-ferredoxin oxidoreductase. Activity of the RG pathway in the acetate oxidation direction could generate one net ATP molecule through the canonical branch, as proposed before [14], whereas the alternative branch through *grd* would yield a null net ATP investment. Even though SAOB are commonly proposed to use an energy-converting hydrogen-pumping hydrogenase such as Ech to create a proton gradient [67], there was no clear evidence for this in *Limnochordia* sp. 5. Instead, both an RNF complex (*rnfCDGEAB*, contig38_50–55) and a protein-translocating transhydrogenase (*pntB,AA1,AA2*, contig44_123–125) were identified and stably expressed. When running the oxidative RG pathway, these complexes could generate a chemiosmotic gradient with simultaneous transfer of electrons to NAD from ferredoxin (RNF) or NADPH (Pnt). These reduced electron carriers are obtained during the oxidative RG pathway by electron bifurcating formate dehydrogenase (contig81_166–167) and the bifunctional methylenetetrahydrofolate dehydrogenase (NADP+) / methenyltetrahydrofolate cyclohydrolase (contig44_269). Among the genes with the strongest upregulation under acetate feeding in this MAG (8 to 16 times) was a putative iron NADH-ferredoxin electron bifurcating hydrogenase (contig38_1068–1071) resembling the reversible Hnd hydrogenase from *Solidesulfovibrio fructosivorans* [68, 69]. NADH could then be regenerated through H_2_ production, transferring electrons to *M. wolfeii*. Further information on gene clusters relevant to energy conservation mechanisms are provided in Supplementary Data 2.

Genes encoding formate dehydrogenase active subunits were found clustered with genes annotated as NADH:quinone oxidoreductases and a putative hydrogenase. Thus, this cluster might represent a periplasmic formate dehydrogenase or formate hydrogenlyase. The mutual up-regulation of *fdh* in *Limnochordia* sp. 5 and *M. wolfeii* points to a cross-feeding of formate from the former to the latter. To test the archaeon’s ability to use formate, we ran a further fed-batch experiment where this compound was the sole carbon source (Supplementary Document 1). Microbial growth displayed a positive dynamics for 4 days, followed by population decay (Supplementary Fig. 4). Such behaviour was expected as formate removed from the medium increased the pH [70]. Moreover, *S. thermophilus* emerged as the main bacterial member, suggesting that this organism could exploit formate for growth or indirectly benefit from archaeal by-products (Supplementary Fig. 4). Indeed, transcriptomics analysis centred on this MAG showed no clear evidence regarding its ability to use the WL, or the RG pathway, for carbon metabolism. However, *fdh* had moderate expression in all the conditions, and amino acid metabolism presented changes in response to varying carbon source ratios (Supplementary Fig. 5).

### Genome-scale mathematical modelling corroborates syntrophic patterns

**Figure 6:**
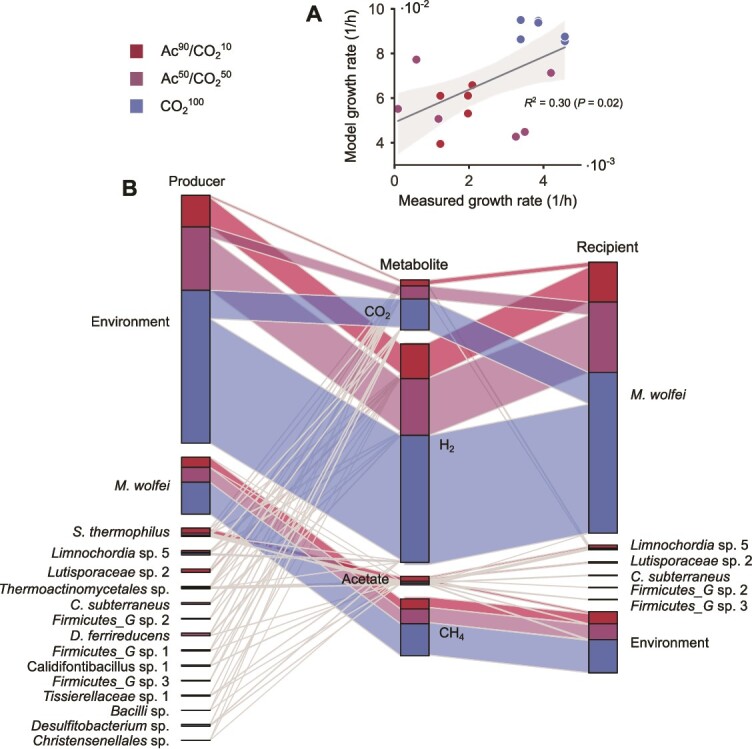
Metabolic activity captured by condition-specific metagenome-scale models of the community. (A) Relationship between measured and modelled community-level growth rate across samples. (B) Exchange fluxes of the key carbon sources, H_2_, and CH_4_ for the modelled community members.

To quantitatively verify the agreement of interspecies formate exchange with our multi-omics data, we turned to metabolic modelling of the microbial consortia. Genome-scale models for the individual taxa were generated, gap-filled, and where necessary, refined starting from MAGs (Methods). Community models were assembled using relative abundances to represent the contribution of individual species across experimental conditions. Depending on the sample and on the number of organisms (from n = 2 up to n = 9), these models accounted for ~3000–12 300 biochemical reactions and 2800–11 000 metabolites. On top of community-level carbon source availability and microenvironmental constraints (Supplementary Data 3), community models were contextualised by metatranscriptomics data integration with CoCo [41] and used to estimate metabolic fluxes and inter-taxon exchanges with ctFBA (Methods) [50]. As a result, we obtained models that included over 90% of mean genomic and transcriptomic abundance and well captured microbial growth across all the carbon source ratios (Pearson *r* = 0.55, *P* value = 0.02—Fig. 6A).

*M. wolfeii* resulted to be the main CO_2_ utiliser, as expected, and released CH_4_ proportionally to the amount of input gases (Fig. 6B). Bacterial partners instead broadly displayed a cooperative behaviour by engaging in CO_2_ cross-feeding at the benefit of the archaeon, which increased in Ac^90^/CO_2_^10^, compensating for its reduced environmental availability. In parallel, a large number of acetate producers and consumers emerged among the low-abundance community members. Acetate was mainly produced by *S. thermophilus*, which also displayed the highest bacterial abundance in Ac^90^/CO_2_^10^.

**Figure 7:**
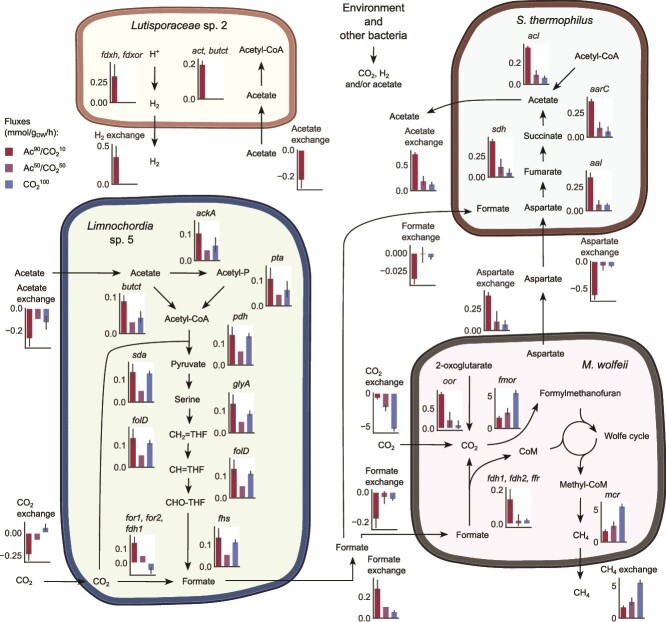
Core metabolic activity and cross-feeding suggested by condition-specific metagenome-scale models. Biochemical fluxes in metagenome-scale models expressed as mmol/g_DW_/h (community dry weight). In exchange pseudo-reactions, negative and positive values denote uptake and release, respectively. Bars and their whiskers represent mean and standard deviation over the replicates, respectively. Abbreviations indicate the following individual reactions: Pyruvate:NAD+ 2-oxidoreductase (CoA-acetylating) (*por*), butanoyl-CoA:Acetate CoA-transferase (*butct*), formate:NADP+ oxidoreductase (*for1*), formate:NAD+ oxidoreductase (*for2*), formate dehydrogenase (*fdh1*), formate dehydrogenase (ferredoxin) (*fdh2*), formate:CoB-CoM heterodisulfide, ferredoxin reductase (*ffr*), 2-oxoglutarate:Ferredoxin oxidoreductase (decarboxylating) (*oor*), formyl-methanofuran oxidoreductase (*fmor*), succinyl-CoA:Acetate CoA-transferase (*aarC*), L-aspartate ammonia-lyase (*aal*), succinate dehydrogenase (menaquinol 7:0 proton) (*sdh*), acetate:CoA ligase (ADP-forming) (*acl*), acetoacetyl-CoA:Acetate CoA-transferase (*act*), ferredoxin hydrogenase (chemiosmosis) (*fdxh*), hydrogen:NAD+, ferredoxin oxidoreductase (*fdxor*).

By more closely analysing flux patterns, formate cross-feeding emerged as a potential link between *M. wolfeii* and *Limnochordia* sp. 5 (Fig. 7). Such an exchange was more elevated in Ac^90^/CO_2_^10^, where the former released over 0.2 mmol/g_DW_/h of formate, partially generated by the RG pathway and partially by the CO_2_ conversion through reactions formate NADP+/NAD+ oxidoreductases (*for1* and *for2*). Formate dehydrogenase reaction (*fdh1*) displayed instead activity in the opposite direction, from formate towards CO_2_, possibly due to cofactor balancing associated with incomplete thermodynamic knowledge, but the net flux resulted in favour of formate generation. Despite the exploitation of external CO_2_, intracellularly produced CO_2_ was also found to allow formate production, suggesting acetate oxidation to be its main driver (Supplementary Fig. 7). *M. wolfeii* was the taxon with the largest formate import, which also increased in absolute value in Ac^90^/CO_2_^10^. Formate was then converted into CO_2_ by the combined action of two formate dehydrogenase reactions (*fdh1* and *fdh2*) and formate CoB-CoM heterodisulfide ferredoxin reductase (*ffr*), with consequent feeding of methanogenesis through the Wolfe cycle. The *ffr* gene also generates coenzyme M (CoM), which is one of the essential cofactors of methanogenesis. In CO_2_^100^, the CO_2_-formate balance in *Limnochordia* sp. 5 shifted towards CO_2_, which served to fuel *por* for pyruvate generation. Thus, formate export decreased, with a concomitant increase in CO_2_ uptake by *M. wolfeii*. Beyond the solution given by ctFBA, the achievable flux space consistent with obtained growth rates show a preference toward formate export rather than import in Ac^90^/CO_2_^10^ (Supplementary Fig. 8).

The role of further bacterial members can be inferred from other metabolites transformed and exchanged at a high rate. In particular, in Ac^90^/CO_2_^10^ the more diverse community established was involved in an intricate cooperative network involving carbohydrates and amino acids. A cross-feeding cascade emerged among the most abundant taxa, departing from *M. wolfeii*, passing through the bacteria, and feeding back into the archaeon (Fig. 7). The latter exported aspartate, which was imported by *S. thermophilus* and fermented into acetate through central metabolism using fumarate, succinate and acetyl-CoA as intermediates. Acetate, already available in the medium, was also absorbed by both *Limnochordia* sp. 5 and *Lutisporaceae* sp. 2. The former used acetate to produce formate as described above, the latter converted that into acetyl-CoA to fuel its growth. The same two taxa were the largest H_2_ producers (*Lutisporaceae* sp. 2 also of CO_2_), thus sustaining archaeal activity. Such a series of cooperative interactions followed a decreasing relative abundance path, with *S. thermophilus* being the most abundant (5.7 ± 0.3%) and *Lutisporaceae* sp. 2 and *Limnochordia* sp. 5 being the second (3.2 ± 0.6%) and the third most abundant (1.8 ± 0.4%), respectively. Hence, these interactions could explain the community structure established in H_2_ and CO_2_ scarcity (Ac^90^/CO_2_^10^). *S. thermophilus* also had the second largest uptake of formate (Fig. 6), coherently with its prevalence over other bacteria when this compound was used as the only carbon source (Supplementary Fig. 4). These patterns thus indicate that such a species can establish a partial competition for formate with *M. wolfeii* in the conditions where this is available.

## Discussion

Microbial syntrophies are recognised to be among the phenomena that define carbon conversion routes in anoxic environments, but their functional bases are not clearly defined, particularly due to the steep scaling of interaction complexity in biologically diverse communities. By drastically reducing the space of possible interactions, this study unveiled several patterns pointing to the syntrophy between *M. wolfeii* and an uncharacterised species, here denoted as *Limnochordia* sp. 5, all revolving around formate cross-feeding via the RG pathway. Although some of these patterns have been reported in synthetic communities built from isolated representatives, our work demonstrated their occurrence in a simplified carbon-fixing community, unearthing the relevance of the RG pathway in this context. This study therefore confirms the advantages of using cultivation strategies for obtaining simplified communities that can serve as tractable models to study inter-species interactions in more realistic settings than with synthetic cultures [16–19].

The identification of the RG pathway as one of the most active is of particular relevance to syntrophy-mediated CO_2_ fixation. Enigmatic observations question the role of the traditionally prominent example of a carbon conversion route associated with syntrophy, the WL pathway, with respect to other known or unknown routes [14]. For example, although involved in the conversion of C1 compounds, it is largely underrepresented in the anaerobic digestion microbiome [15]. The RG pathway has recently been discovered to allow autotrophic growth in *Desulfovibrio desulfuricans* [71] and *Clostridium drakei* [72] and, given its partial overlap with the WL, it represents a plausible candidate for syntrophy mediation. Here, we showed that this pathway is fully present and expressed in an uncharacterised organism, *Limnochordia* sp. 5, that is one of the two main bacteria consistently growing in co-presence with *M. wolfeii* under varying CO_2_ concentration regimes. Our results indicate that, similarly to the bidirectional usage of the WL pathway by other species [5, 13], *Limnochordia* sp. 5 may exert both acetate oxidation and homoacetogenic activity through the RG pathway. Such a bidirectionality could confer a high versatility upon *Limnochordia* sp. 5 and could therefore explain the widespread presence of the MAG in anaerobic digesters, along with metabolic pathways conferring a high phenotypic plasticity [15]. Further, our results support a fundamental role of formate for syntrophic CO_2_ fixation. Beside *M. wolfeii*, other hydrogenotrophic methanogens encoding the formate dehydrogenase genes may be able to respond to CO_2_ shortages by exploiting the syntrophic relation with *Limnochordia* sp. 5 and potentially other species for biomass accumulation. Formate has indeed been observed to be exchanged in combination with H_2_ and has been suggested to be the preferred electron carrier in aqueous environments [4], although such a preference might depend on other environmental factors like ammonia concentration [73]. Future experiments might confirm the nature of such exchanges by tailored biochemical approaches such as isotope tracking. Further, what remains to be elucidated is how a bidirectional RG pathway would allow energy conservation. The presence of multiple hydrogenases and membrane-associated gene clusters may suggest the use of alternative ion gradients like in other anaerobic microorganisms (Supplementary Data 2) [74]. In future studies, the thermodynamic balance will need to be evaluated in adequate experimental conditions.

The role of *S. thermophilus* adds one intriguing element to the possible mechanism underlying community assembly during CO_2_ fixation. Amino acid exchange has recently been under the lens of numerous studies as a prominent mode for syntrophism [4, 14, 75], and their function has been linked to resolving auxotrophies [4, 10, 76]. The results of this study point to amino acid fermentation as a driving force for SAOB activity. In our experiment acetate was externally provided but we hypothesise that, if absent, its production by *S. thermophilus* could generate a synergistic metabolic circuit perpetuating hydrogenotrophic methanogenesis. This find thus suggests a role of amino acids in CO_2_-fixing microbiomes that may deserve further attention.

This work revealed a scenario where phenotypic plasticity allows the sustenance of a dominant subset of species under carbon source shift. The predicted interactions and their strength are expected to change under different environmental conditions. For example, phenotypic plasticity and associated variation in ecological interactions depend on the relative abundance of the involved species and strains [77, 78]. Here, *M. wolfeii* was by far the dominant member, whereas in natural settings methanogens comprise only a small fraction. Thus, even though spatial or temporal partitioning could locally create methanogen-prevalent communities, it is generally uncertain how frequently the presented interactions could arise in natural situations. The selective conditions applied, initial community structure, and stochastic phenomena are likely to have jointly contributed to the establishment of the described cooperating microbial system. For instance, in this study the distribution of single-nucleotide variants over the course of the simplification process suggests that their accumulation might have played a role (Supplementary Table 5), but in general cooperation strategies are expected to depend on all these factors. Moreover, when numerous bacterial species have a relevant abundance, higher-order interactions are expected to arise, differently from simplified communities. These questions could be addressed by applying complementary techniques such as isotope labelling. Nevertheless, community simplification remains a valuable approach to pinpoint key interactions whose flexibility can later be tested.

## Supplementary Material

Unlinked_Supplementary_Document_1_wraf017

Supplementary_Data_1_wraf017

Supplementary_Data_2_wraf017

Supplementary_Data_3_wraf017

## Data Availability

Raw metagenomic and metatranscriptomic data generated in this study have been deposited in NCBI under BioProject number PRJNA999073. The MAGs, transcriptomes, and full data and code used in genome-scale metabolic modelling are available at https://github.com/gzampieri/syntrophy/.
