# Supplementary material for: Core cooperative metabolism in low-complexity CO2-fixing anaerobic microbiota
Source: ISME J. 2025 Feb 2;19(1):wraf017. doi: 10.1093/ismejo/wraf017 (PMC11844248; doi:10.1093/ismejo/wraf017)
Supplement: Unlinked_Supplementary_Document_1_wraf017 [file unlinked_supplementary_document_1_wraf017.docx]

Core cooperative metabolism in low-complexity CO_2_-fixing anaerobic microbiota

Supplementary information

Guido Zampieri^a^*, Davide Santinello^a^*, Matteo Palù^a^, Esteban Orellana^a^, Paola Costantini^a^, Lorenzo Favaro^b,c^, Stefano Campanaro^a^^, Laura Treu^a^

* These authors contributed equally to this work

^ Corresponding author: stefano.campanaro@unipd.it

a: Department of Biology, University of Padova, Via U. Bassi 58/b, 35121 Padova, Italy

b: Department of Agronomy, Food, Natural Resources, Animals and Environment (DAFNAE), University of Padova, Agripolis, Viale dell'Università 16, 35020 Legnaro, PD, Italy

c: Department of Microbiology, Stellenbosch University, Private Bag X1, Matieland 7602, South Africa

## File descriptions

**Supplementary Document 1.docx:** Supplementary methods, supplementary results, Supplementary Figure 1-5, and Supplementary Table 1-4.

**Supplementary Data 1.xlsx:** Full quality and taxonomic information on metagenome-assembled genomes and associated relative abundances.

**Supplementary Data 2.xlsx:** Normalised fragments per kilobase for *M. wolfeii*, Limnochordia sp. 5 and *S. thermophilus* and results of differential expression analysis comparing the expression of the same MAGs in the Ac^90^/CO_2_^10^ condition against CO_2_^100^.

**Supplementary Data 3.xlsx:** Genome-scale metabolic model information including community model structure, experimental constraints, and details on the reactions of *M. wolfeii*, Limnochordia sp. 5 and *S. thermophilus*.

## Supplementary methods

### Microbial community simplification

Several inoculation steps (n=6) were carried out, each time diluting the cultures obtained previously by a factor proportional to the working volume. Upon reaching a higher methane production compared to the previous generation, each culture was reinoculated in fresh BA medium. Culture volume was gradually increased throughout reinoculations to a final volume of 300 mL. Cultures were fed on CO_2_ and H_2_ on a 1:4 volumetric ratio every five days, increasing feedstock volume according to liquid culture volume (Supplementary Table 1).

### Analysis of community simplification

Diversity for the trickle-bed reactor microbiome was calculated on a previously obtained shotgun sequencing dataset of the reactor (Sequence Read Archive accession SRX9933233) (1). Preliminary investigation of microbial consortium composition during simplification (at the fourth generation) was conducted through sequencing of the V4 hypervariable region of 16S rRNA gene. To calculate the diversity, a sample was collected from the final (sixth) generation before pre-adaptation and analysed with the same shotgun sequencing methodology as the samples from the experimental phase.

Amplicons were processed and sequenced with primers 515 F and 806 R by using the paired-end Illumina MiSeq platform at the sequencing facility of the Department of Biology, University of Padova (Padova, Italy). Data analysis was performed with CLC Workbench (v8.0.2) using the microbial genomics module plug-in (QIAGEN Bioinformatics, Germania). Standard quality filters were applied prior to operational taxonomic unit (OTU) clustering, taxonomic classification (GreenGenes v13.8) (2) and estimation of relative abundance. Taxonomic assignment of most abundant OTUs was manually checked by using BLAST and the 16S ribosomal RNA gene database (Bacteria and Archaea), comparing the resulting taxonomy with that obtained by CLC.

### Gram determination and viability assays

To determine microbial morphology, Gram staining was performed by adopting a modified version of a previous protocol (3). Briefly, 50 𝜇L aliquots diluted in phosphate buffered saline (PBS) were deposited on a Petri dish let to dry and fixed by heating. Samples were then stained with crystal-violet (1.24 g in 100 mL of water) for one minute, washed with water, and treated with Lugol (0.33 g iodine and 0.67 g potassium iodide in 100 mL water) for another minute. Upon a second wash, 96% ethanol was used for 30 seconds to remove the stain and safranin (0.01 g in 10 mL of methanol) was used for two minutes for re-staining. Finally, the samples were washed again.

Cell viability was evaluated by fluorescein diacetate and propidium iodide (FDA-PI) double staining assays (4). Such assays were run in vivo using a PDA-PI and PBS mixture solution, without heat fixation. After centrifuging (8000 rpm, two minutes, 25°C) and removing the supernatant, samples were resuspended in 100 𝜇L of PBS, newly centrifuged (8000 rpm, two minutes, 25°C), supernatant-filtered, and finally resuspended in 100 𝜇L of FDA-PI solution. After five minutes of ice incubation in the dark, the samples were deposited on a slide and immediately utilised for microscopy analysis.

Both Gram stain and cell viability were assessed with a DM5000B (Leica, Germany) fluorescence microscope, using LAS v4.12. A 490 nm and a 520 nm filter were applied for absorption and emission light in cell viability analysis, respectively.

### Experimental setup

Inoculum seeding as well as synthetic medium and oligo-nutrient addition were performed under anoxic conditions inside a MB200B inert glove box (MBRAUM, Germany) filled with a N_2_ atmosphere. After nutrient addition, the medium was reduced with Na_2_S and the reactors were sealed tight with bromobutyl rubber stoppers and caps. Reactors were then sparged with N_2_ gas for 10 minutes and kept at 55°C for one hour. The reactors were operated at 55±2°C and kept under constant linear shaking (115 rpm) for the duration of the experiment (14 days). pH was maintained at values equal or higher to 7.00 by NaOH (1M) adjustment.

### Amino acid quantification

Amino acid detection and quantification was performed by UPLC-MS/MS at Italiana Biotecnologie s.r.l. (Montebello Vicentino, Italy) with the AccQ•Tag Ultra method (Waters Corporation, Milford, USA). Sample supernatant was derivatized prior to column injection using Waters AccQ•Tag Ultra Derivatization Kit according to manufacturer’s protocol modified to include 10 μl of norvaline as internal standard and reducing the amount of borate buffer from 70 to 60 μl.

### Feeding regimen of secondary cultures

A second set of triplicate scaled down fed-batch reactors (indicated as FOR, AA, BES, BES/AA) was used. The reactors consisted of 120 mL glass bottles (42 mL working volume) filled with a basal anaerobic medium, with a yeast extract addition (0.2 g/L final concentration). 2-bromoethanesulfonate (BES) was added in BES and BES/AA groups bottles at a final concentration of 20 mM as described by Salvador and colleagues (5). Moreover, amino acid solutions for aspartic acid, valine, and leucine were added to the bottles from the AA and BES/AA group in a final concentration equal to inoculum-free synthetic medium. Without accounting for the yeast extract, the concentration for aspartic acid, valine, and leucine were 19.1 μM, 32.5 μM, and 26.7 μM, respectively.

Bottles from groups AA, BES, and BES/AA were subject to a stoichiometric 1:4 CO_2_ and H_2_ addition. FOR group bottles feeding was supplied as formic acid (100% w/w). Gas feeding was performed daily, whereas formic acid addition was performed every four days.

Samples from FOR, AA, BES, BES/AA triplicate reactors were collected after 14 days at the fermentation end-point. For these samples, DNA was extracted using the DNeasy PowerSoil Pro Kit (QIAGEN GmbH, Hilden, Germany) following manufacturer’s protocols with an additional step of 2 mL of Phenol:Chloroform:Iso-amyl alcohol 25:24:1 pH 8 (Sigma-Aldrich, St. Louis, MO, USA) performed to increase sample purification.

### Targeted metabolic pathway reconstruction

Completeness of the Wood-Ljungdahl (WL) pathway and the reductive glycine (RG) pathway in each MAG was determined by comparing the KOs predicted from genome annotation with those listed in module M00377 (reductive acetyl-CoA pathway) of KEGG. A manual refinement of the list expanded the selection to include alternative formate dehydrogenases and the *pta*-*ackA* module. Presence of KEGG modules blocks was assigned as follows: for reactions catalysed by a single gene, the block for the corresponding gene was marked if the KO was found. For reactions catalysed by enzymatic complexes, the block was assigned if 50% or more of the constituent subunits were found. Exceptions were as follows: the *fdh* block (representing ECs 1.17.1.10, 1.17.1.9, 1.17.1.11) was considered present if any of, the active subunits of formate dehydrogenase (NADP+) (K05299, K15022) or the major subunit of formate dehydrogenase (K00123) was found, or alternatively 50% of the electron-bifurcating formate dehydrogenase; for the POR block, a match for *por* (K03737) was considered as alternative to the *porA-D* subunits, according to the formula of KEGG module M00307. Manual refinement of protein function was performed via BLASTp, eggNOG-mapper, InterPro and available literature. Representations of reconstructed metabolic pathways were created with Biorender.com.

### Genome-scale model refinement

The draft models for *M. wolfeii* and *Limnochordia* sp. 5 were manually refined before gap-filling based on experimental data and biological hypotheses. Specifically, *M. wolfeii* was provided with transporters for those amino acids that were observed to be consistently consumed in the mixed cultures (Supplementary Fig. 1) using significant BLASTp hits returned by gapseq when defining respective gene-protein-reaction (GPR) rules. Given the prevalence of *M. wolfeii*, the archaeon was arguably an active participant in such consumption. Only hits having a bit-score greater than 150 were considered, except for those amino acids without hits above such threshold, for which a minimum bit-score of 130 was used. In *Limnochordia* sp. 5, the WL and RG pathway were reviewed ensuring that all the reactions were present, their GPR was consistent with functional annotation, and their directionality allowed reversed activity.

### Community model and experimental constraint definition

A microbial community model was created for each sample by using Micom (6). The inclusion of microbial taxa in community models was based on the relative abundances of corresponding MAGs and transcriptomes. For the MAGs, the relative abundances were obtained with CheckM from their read coverage (7). For the transcriptomes, an analogous coverage was defined using the total read counts (log_2_-rescaled) and the total length of gene sequences in a MAG, following the same principles. Hence, given a MAG $i$, its transcriptional coverage $c_{i}$ was calculated from the total number of RNA reads mapping on its gene sequences and from the sum of its gene sequence length $l_{i}$ as follows:


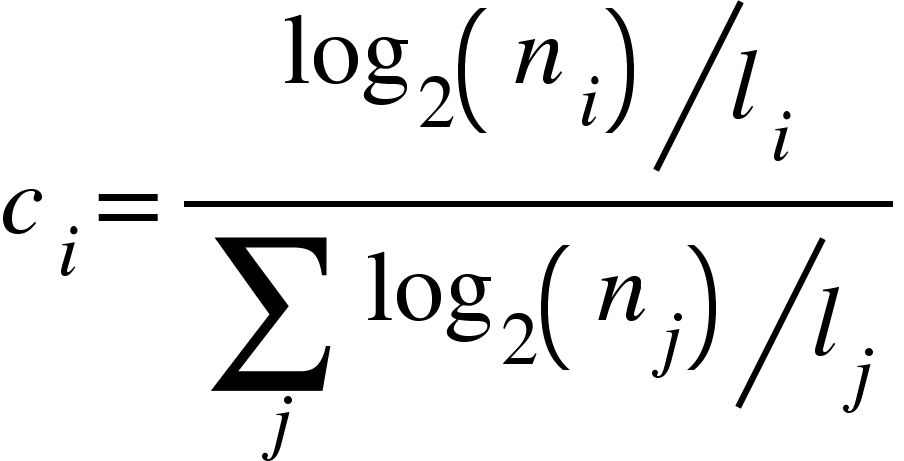


where the summation at the denominator is over all the MAGs. “Multi-omic relative abundances” were then obtained by multiplying MAG and transcriptome relative abundances and re-normalising the values to 1. Any taxon with multi-omic abundance equal or greater than 0.5% in a given sample was included in the community model for that sample.

In Micom, cooperative trade-off flux balance analysis (ctFBA) was used to model microbial growth rates. Denoting any community member with $i$, let us define the associated stoichiometric matrix with ***S****_i_*, metabolic reaction flux vector with ***v****_i_*, relative abundance *a_i_*, and growth rate with *𝜇_i_*. By introducing an external compartment representing the environment and enclosing all the community members, the exchanges between any individual member *i* and the environment can be denoted as ***v****_i_^ex^* whereas the exchanges between the entire community and the environment can be identified by ***v*** *^m^*. Together, all these fluxes are limited by the following community constraints:


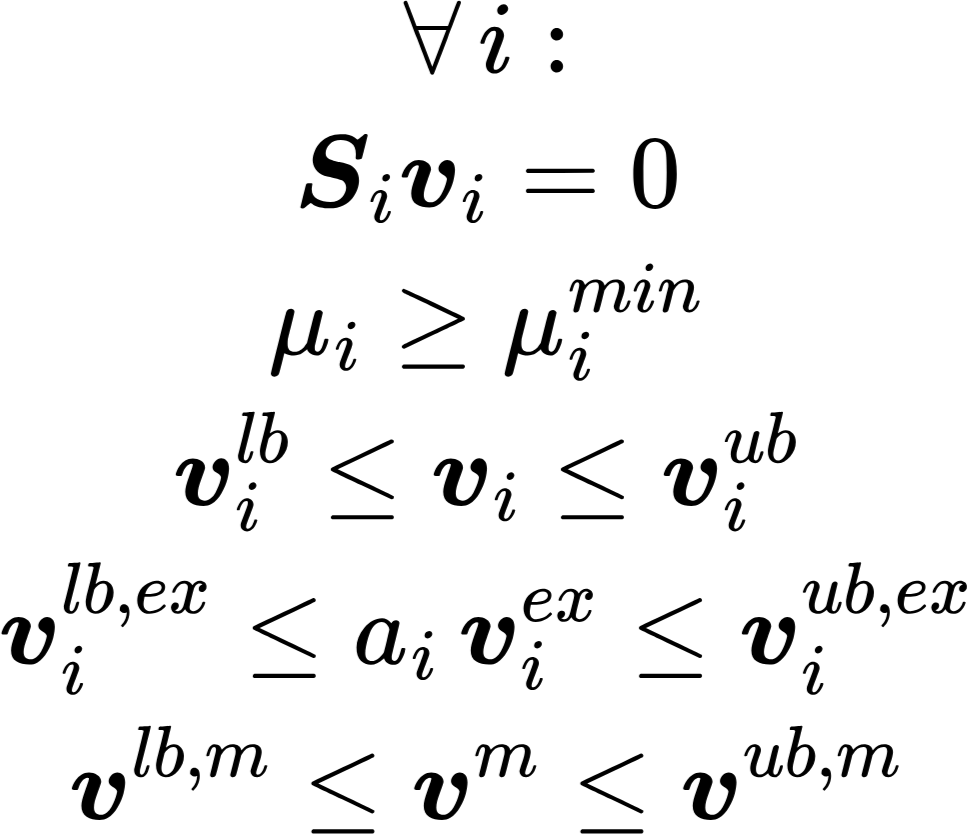


Using these constraints, ctFBA is formulated as follows:


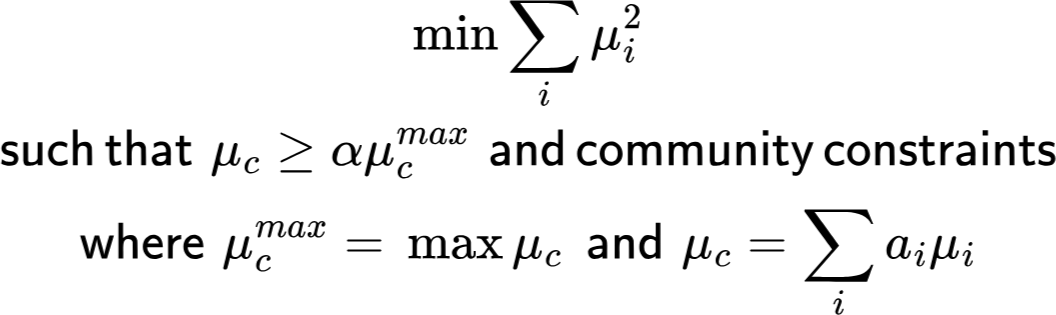


Here, *𝜇_c_* represents the growth rate of the entire community and 𝛼 is an arbitrary trade-off parameter between 0 and 1. Thus, ctFBA first calculates the maximum community growth rate *𝜇_c_^max^* and next minimises the L2 norm of community-member-specific growth rates. This strategy allows obtaining balanced and unique growth rates for individual taxa that are consistent both with their maximisation and the optimisation of *𝜇_c_*.

Community-level constraints were set based on the carbon sources and electron donors provided (acetate, H_2_, and CO_2_) and the amino acid concentrations in the cultures. Gas volumes and amino acid concentrations on the day prior and after each biological sampling point were used to calculate their net consumption or production rates r. Dry weight estimates and total culture volumes were used to obtain rates expressed in mmol/g/h. Dry weights at the biological sampling points were obtained by using final dry weights and OD curves assuming a linear relationship between the two quantities. Rate errors 𝛥r were calculated by uncertainty propagation as follows. For all the biochemical measurements, a 20% random error was taken as a maximum uncertainty and for UPLC-MS/MS measurements a systematic uncertainty of 20 𝜇M was also assumed given the low amino acid concentrations (8). Dry weight errors were obtained by propagation from OD uncertainties. Random and systematic errors were propagated independently. Lower and upper bounds were set as r ± 3∙𝛥r.

### Additional bioinformatic analyses

Microbial replication rates were estimated using CoPTR 1.1.6 (9) starting from high-quality MAG sequences and filtered short reads and with default parameters. The obtained values are log_2_ peak-to-trough ratios (PTRs) of the sequencing coverage along any microbial genome, which have been shown to reflect condition-specific growth rates. Single-nucleotide variants (SNVs) accumulated over the community selection process were estimated using inStrain 1.7.1 (10) with --min_mapq 2 --min_read_ani 0.98 --min_genome_coverage 1. The resulting SNVs were filtered requiring a minimum position coverage of 10.

## Supplementary results

### Methanogenic pathway reconstruction for *Methanothermobacter wolfeii*

As described in the main text, *M. wolfeii* was the only archaeon identified in the microbiome and, thus, the sole responsible for methanogenesis. KEGG Orthology (KO) classifications predicted for the corresponding MAG were screened for KOs listed in KEGG module M00567 (Methanogenesis, CO2 => methane). Manual refinement by BLASTp of predicted protein sequences against the non redundant (NR) database of NCBI was necessary to confirm assignment of subunits to either isozyme complex. The MAG had genes encoding for the complete hydrogenotrophic methanogenesis pathway via the archaeal-type Wood-Ljungdahl (WL) pathway. This harboured isozymes previously identified in the reference genome SIV6 (11), in particular:

- formylmethanofuran dehydrogenase isozymes Fwd (*fwdHFGDACB*) and

Fmd (*fmdBCE*);

- methylenetetrahydromethanopterin dehydrogenase isozymes Mtd and

Hmd;

- methyl coenzyme-M reductase isozymes MCR I (*mcrBDCGA*) and MCR II

(*mrtBDGA*).

The MAG encoded the archaeal CODH/ACS (*cdhABCDE*) as a gene cluster, whereas other five genes for *cdhAB* constituted a cluster on their own (CdhAB 2). A reconstruction of the identified *M. wolfeii* methane metabolism is presented in the main manuscript.

Completeness of the hydrogenotrophic methanogenesis pathway confirms what is reported in the literature regarding the MAG role and its capability of utilising H_2_ and CO_2_. In addition, the presence of the CODH/ACS suggests the ability of *M. wolfeii* to fix CO_2_ to acetyl-CoA, probably for biomass accumulation. Not all hydrogenotrophic methanogens possess this trait and other species are instead known to uptake acetate for biomass purpose. The CODH/ACS complex shapes several possible interactions of *M. wolfeii* with the community by allowing growth on pure H_2_ and CO_2_: homoacetogenic bacteria are competitors as they convert CO_2_ to acetate, conversely syntrophic acetate-oxidising bacteria (SAOB) are largely benefitting the methanogen by releasing H_2_ and CO_2_. Additionally, H_2_/CO_2_ removal by the methanogen favours SAOB by shifting the equilibrium of acetate oxidation.

### Effect of amino acid supplementation

Despite not being the main objective of the study, we also tested the role of bacterial taxa in the light of other mutualistic interactions. In particular, amino acids have long been hypothesised to be at the centre of inter-kingdom mutualism and, recently, evidence has accumulated towards exchanges resolving bacterial auxotrophies (12). In fact, amino acids were verified to be rapidly consumed in all the conditions (Supplementary Fig. 2). We thus performed a secondary fed-batch experiment using the same inoculum, where CO_2_ was the sole carbon source and three distinct conditions were established by adding the following compounds: (i) aspartic acid, valine, and leucine (AA); (ii) 2-bromoethanesulfonate (BES); and (iii) both together (BES/AA). The experiment aimed to verify whether these amino acids could allow the increase of bacterial taxa obviating the suppression of the archaeal partner due to BES addition. As a result, several bacterial members increased in relative abundance in BES/AA compared to the other conditions, such as *Desulfotomaculum ferrireducens*, *Calidifontibacillus* sp., and *Firmicutes G* spp., possibly in agreement with the idea that the archaeal species can support selected microbes through amino acid exchange (Supplementary Fig. 2). Alternatively, *M. wolfeii* might compete for these amino acids with the bacteria, given their higher abundance both in the BES and BES/AA conditions.

### Analysis of *fhs* expression across bacterial MAGs

By using RNA-seq data we inspected the activity of the WL and RG pathways across experimental conditions (Fig. 4B). Formate-tetrahydrofolate ligase (FTHFS), encoded by the *fhs* gene, catalyses the second-to-last step of both the WL and RG pathway, i.e., the hydrolysis of formyl-THF with the release of formate and generation of one ATP. Therefore, FTHFS is a key enzyme in the proposed acetate oxidation pathways as it accomplishes the dual role of electron transfer to the hydrogenotrophic partner and energy conservation via substrate-level phosphorylation. Indeed, *fhs* is the only consistently identified gene in the genome of known SAOB, and its expression has been used as a marker for determining the syntrophic acetate oxidation activity (13). Therefore, we analysed *fhs* expression over the high-quality MAG fraction of the community (Fig. 4B). As both abundance of the MAG and expression of the gene determine the contribution of each MAG to the total enzyme activity in the microbiome, non-normalised FPK values were considered (Methods).

The scarcity of CO_2_ was associated with a shift in the species with the highest *fhs* activity. The most abundant bacterial MAG in Ac^90^/CO_2_^10^, *S. thermophilus*, did not show an analogous dominance in terms of *fhs* expression. In contrast, *Limnochordia* sp. 5 displayed a high *fhs* expression level both in CO_2_^100^ and Ac^90^/CO_2_^10^. Among other taxa, Tissierellaceae sp. 1 had the largest *fhs* activity in CO_2_^100^ and a negligible activity in Ac^90^/CO_2_^10^, where its presence was also lower. Accounting for changing MAG abundance, normalised FPK counts indicated a nine-fold downregulation in *fhs* expression in Ac^90^/CO_2_^10^ communities. Based on these findings, Tissierellaceae sp. 1 could be hypothesised to be a H_2_-utilising bacterium, although it did not harbour a complete WL pathway, making it a competitor for the archaeon. Besides, Tepidimicrobiaceae sp. 1 had the highest *fhs* activity of the community in high acetate. According to the normalised FPK counts, expression of *fhs* doubled in Ac^90^/CO_2_^10^ as compared to CO_2_^100^, whereas mixed feeding resulted in partially increased expression, suggesting a regulation process based on the amount of acetate. Therefore, the presence and activity of the complete RG pathway suggests that Tepidimicrobiaceae sp. 1 can be hypothesised to be a SAOB, despite lacking the carbonyl-branch of the WL pathway. More direct evidence is needed to further clarify the metabolic path followed by acetate in this species.

### *Sphaerobacter thermophilus* functional activity

*S. thermophilus* showed high affinity for acetate feeding, with a mean relative abundance of 5.7% in Ac^90^/CO_2_^10^. Principal component analysis (PCA) showed separation of transcriptomics profiles based on the carbon source ratio (Supplementary Fig. 5). In the principal component plane, clustering patterns suggest a more defined behaviour in Ac^90^/CO_2_^10^ and CO_2_^100^, whereas the Ac^50^/CO_2_^50^ cluster is more widely spread. Even though these patterns pertain to *S. thermophilus* transcriptome, they might also reflect more heterogeneous inter-species interactions in the mixed condition.

Analysis of WL and RG pathway genes showed little difference in expression between Ac^90^/CO_2_^10^ and CO_2_^100^ conditions, with the log_2_ fold change for most genes within one (Supplementary Fig. 5). Yet, an overall downregulation of the WL/RG pathway was apparent with acetate feeding: three of the annotated acetyl-CoA synthetase genes (averaged into ACSS 1) were downregulated, whereas ACSS 2 followed an opposite trend. Glycine hydroxymethyltransferase (*glyA*) was downregulated. *gcvPB* and *gcvT* were noticeably downregulated, although their lowest expression was with mixed feeding, suggesting a more complex regulation than based on the level of acetate in the medium alone. *fol*D, *fhs* and *fdh* expression was largely stable.

It is possible that the shifts in expression of *gly*A and RG pathway genes are linked to the changes observed in amino acid transporters. As the activity of *M. wolfeii* and other bacteria in the community results in different amino acids being released in the medium, *S. thermophilus* might be responding to these changes in its environment by varying its amino acid uptake ability, and generate energy via amino acid hydrolysis. The increased expression of ACSS in CO_2_^100^ might be required for synthesis and extrusion of acetate from pyruvate with generation of 1 ATP molecule.

## Supplementary figures

**
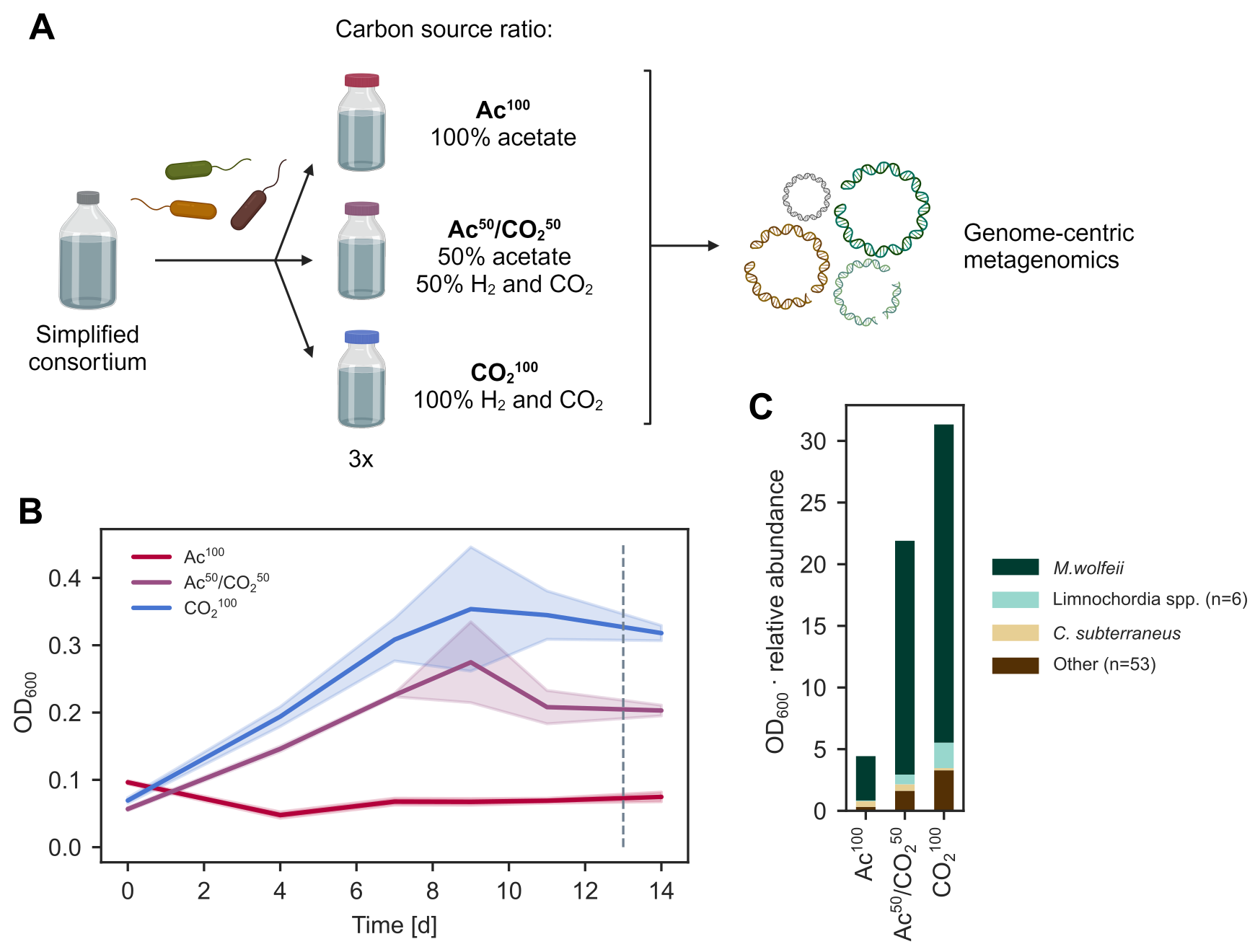
**

**Supplementary Figure 1: Preliminary adaptation to different carbon source ratios.** (A) Upon simplification, the consortium was fed with a minimal medium whose carbon source was either only acetate, mixed acetate and CO_2_ and only CO_2_. These communities were characterised through DNA shotgun sequencing. (B) Growth dynamics of the microbial communities. The vertical dashed line indicates the sampling point. (C) Absolute microbial abundances obtained by multiplying optical density and relative abundances obtained from DNA shotgun sequencing.


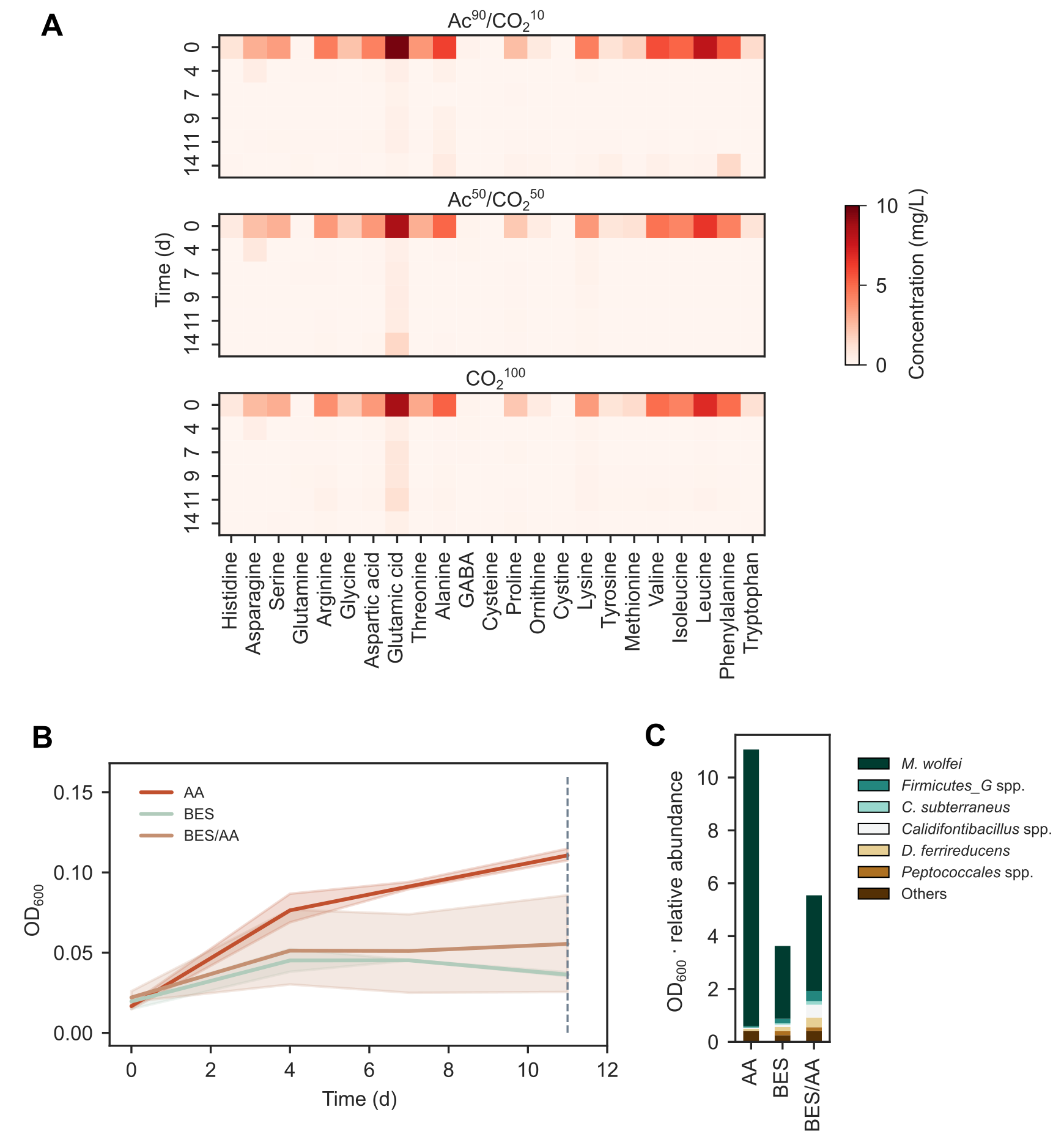


**Supplementary Figure 2: Amino acid utilisation in the simplified community.** (A) Amino acids were rapidly consumed in the early growth phase with all the carbon source combinations. (B) The effect of extra amino acid supplementation was tested, verifying whether this could restore bacterial abundance upon methanogenesis inactivation with BES. (C) Microbial growth dynamics when supplementing amino acids, BES, and both. The vertical dashed line indicates the sampling point. (D) Absolute microbial abundances obtained by multiplying optical density and relative abundances obtained from DNA shotgun sequencing at day 11.


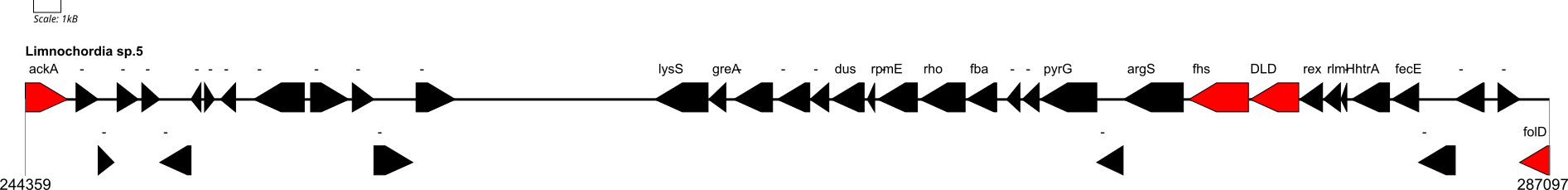


**Supplementary Figure 3: Genomic localisation of RG pathway genes in *Limnochordia* sp. 5.** Gene sequences not associated with known gene products are left unlabelled.


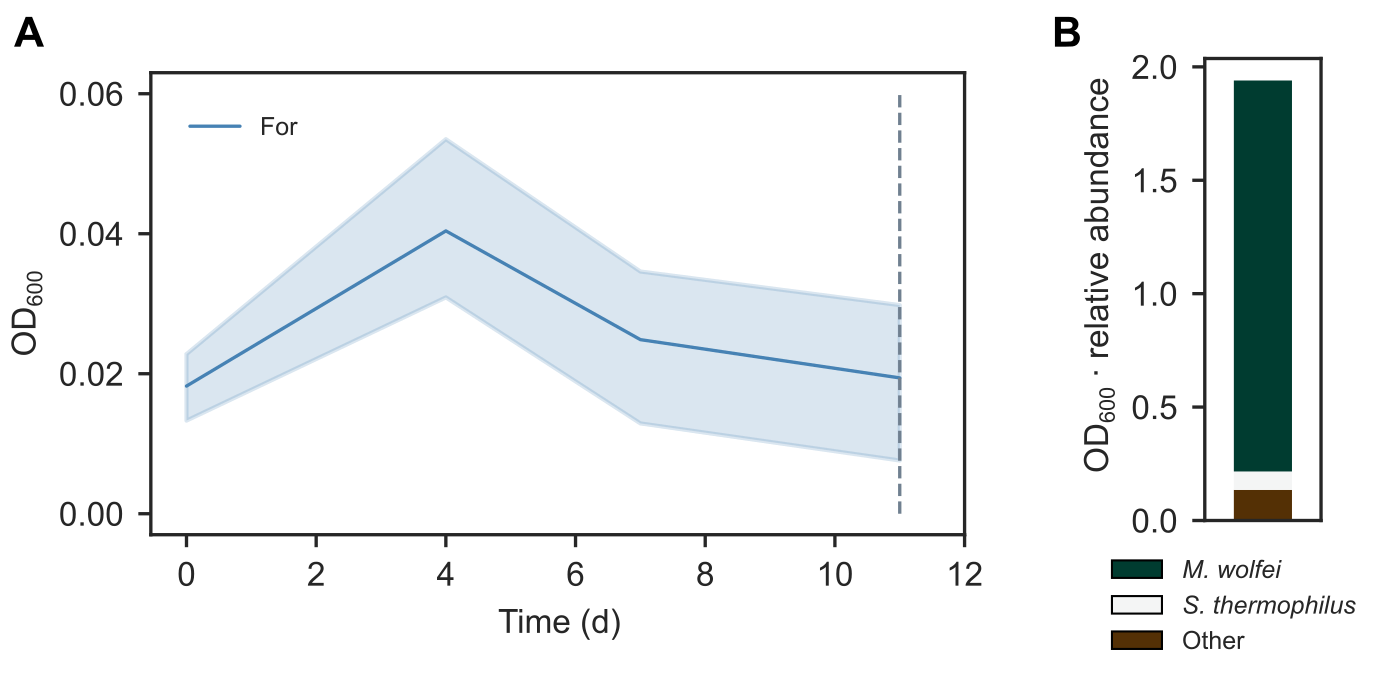


**Supplementary Figure 4: Effect of formate feeding.** (A) Microbial growth dynamics when fed uniquely with formate as a carbon source. The vertical dashed line indicates the sampling point. (B) Absolute microbial abundances obtained by multiplying optical density and relative abundances obtained from DNA shotgun sequencing at day 11.


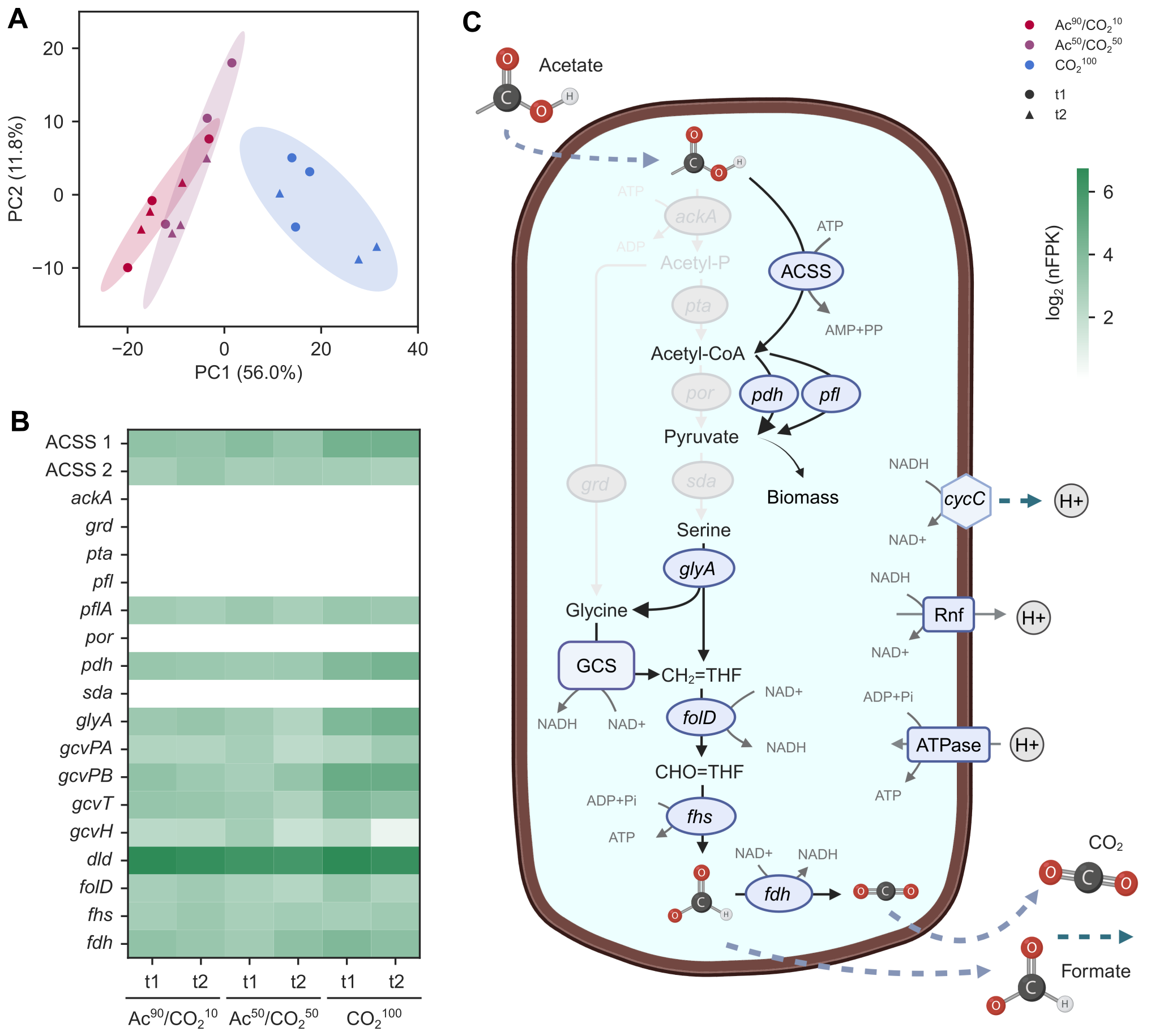


**Supplementary Figure 5: Transcriptional activity of *S. thermophilus* under distinct feeding regimes.** (A) Principal component representation of *S. thermophilus* transcriptomic activity across samples. (B) Mean gene expression of genes for the RG pathway. (C) Diagram of carbon assimilation, acetate oxidation and homoacetogenesis pathway in *S. thermophilus* illustrating the genes and reactions identified or absent in the MAG.


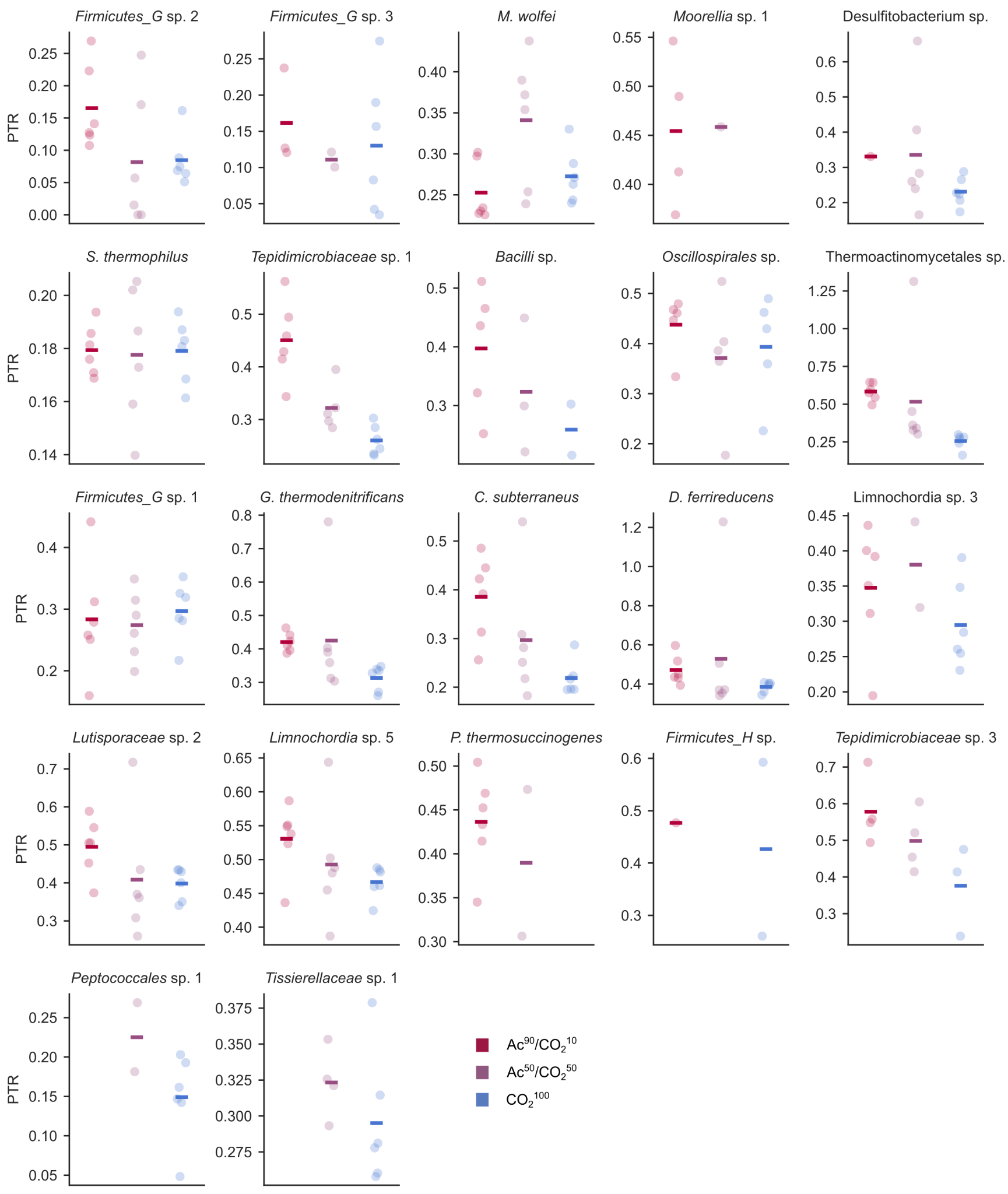


**Supplementary Figure 6:** Microbial replication rates across carbon source ratios estimated as log_2_ PTRs from MAGs.


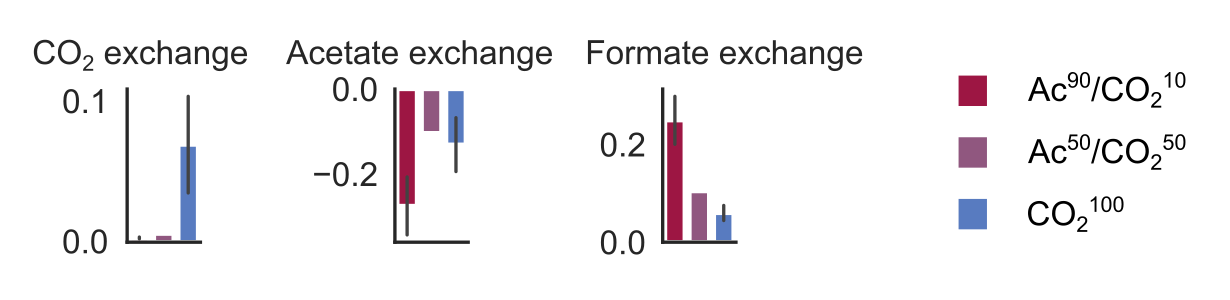


**Supplementary Figure 7:** Main exchange flues in *Limnochordia* sp. 5 when constraining CO_2_ uptake to be null.

##
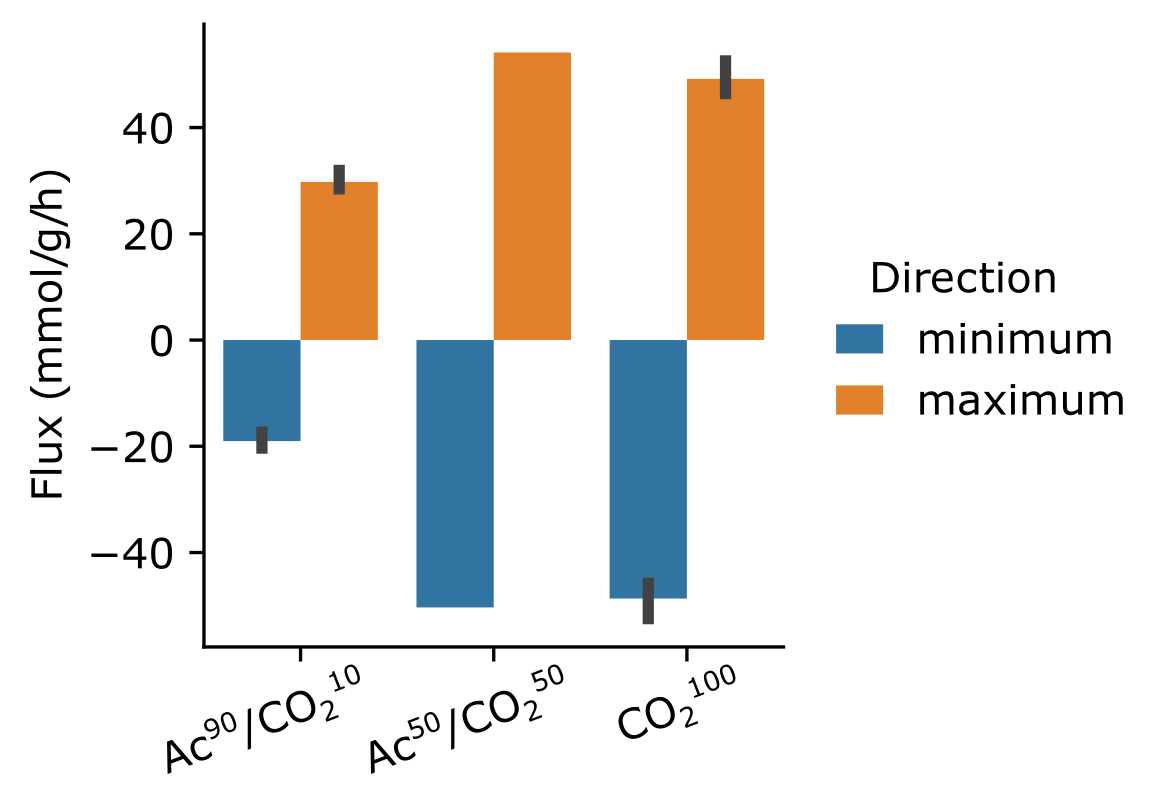


**Supplementary Figure 8:** Flux span for formate exchange in *Limnochordia* sp. 5 when constraining microbial growth rates to those obtained by ctFBA.

## Supplementary tables

**Supplementary Table 1:** Stages of community simplification and enrichment.

| **Generation** | **Culture volume (mL)** | **Reactor volume (mL)** | **Injected CO_2_ (mL)** | **Injected H_2_ (mL)** |
| --- | --- | --- | --- | --- |
| 1^st^ to 4^th^ | 30 | 120 | 12 | 48 |
| 5^th^ | 300 | 1000 | 125 | 500 |
| 6^th^ | 300 | 2000 | 400 | 1600 |
| Final pre-adaptation | 350 | 1000 | 150 | 600 |

**Supplementary Table 2:** Microbial taxonomy of the consortium under selection at generation four, obtained by 16S rRNA gene amplicon sequencing.

| **OTU** | **Relative abundance (%)** |
| --- | --- |
| g__Methanothermobacter, 369183 | 59.70 |
| f__Peptococcaceae, 262551 | 21.05 |
| g__Desulfotomaculum, 365127 | 1.34 |
| OTU-0002 | 1.12 |
| OTU-0009 | 1.12 |
| OTU-0037 | 1.04 |
| o__MBA08, 1121084 | 0.87 |
| OTU-0004 | 0.80 |
| OTU-0003 | 0.68 |
| f__[Tissierellaceae], 689806 | 0.61 |
| OTU-0011 | 0.54 |
| OTU-0010 | 0.53 |

**Supplementary Table 3**: Feeding regimes of the main reactors and approximate methane production rates. Gas over-pressure and non-homogeneous mixing in fed-batch reactors lead to an underestimation in the production rates for Ac^50^/CO_2_^50^ and CO_2_^100^.

| **Reactor group** | **Feeding (mmol/d)** | **CO_2_ to total carbon molar ratio** | **CH_4_ production at t1 (mmol/g_DW_/h)** | **CH_4_ production at t2 (mmol/g_DW_/h)** |
| --- | --- | --- | --- | --- |
| Ac^90^/CO_2_^10^ | 4.84 CH_3_COOH + 0.67 CO_2_ + 2.70 H_2_ | 0.12 | 0.63±0.10 | 0.88±0.26 |
| Ac^50^/CO_2_^50^ | 2.76 CH_3_COOH + 2.76 CO_2_ + 11.04 H_2_ | 0.50 | 2.60±1.42 | 1.62±1.23 |
| CO_2_^100^ | 5.52 CO_2_ + 22.07 H_2_ | 1.00 | 2.87±0.33 | 0.62±0.78 |

**Supplementary Table 4:** Feeding regimes of the secondary reactors.

| **Reactor group** | **Feeding** |
| --- | --- |
| AA, BES, BES/AA | 0.96 mmol CO_2_ + 3.82 mmol H_2_ |
| FOR | 1.33 mmol HCOOH |

**Supplementary Table 5:** Single-nucleotide variant (SNV) distribution in the main community members during the simplification process. These define the point differences between the MAGs obtained from the DNA at the end of simplification and the DNA collected during simplification.

| **MAG** | **Generation** | **Relative abundance (%)** | **SNVs** |
| --- | --- | --- | --- |
| *M. wolfeii* | 3^rd^ | 62.0 | 1,807 |
|  | 5^th^ | 97.6 | 1,921 |
| *S. thermophilus* | 3^rd^ | 19.2 | 5,045 |
|  | 5^th^ | <0.1 | 0 |
| *Limnochordia* sp. 5 | 3^rd^ | 4.4 | 7,084 |
|  | 5^th^ | 0.3 | 1,262 |

## References

1. Tsapekos P, Treu L, Campanaro S, Centurion VB, Zhu X, Peprah M, et al. Pilot-scale biomethanation in a trickle bed reactor: Process performance and microbiome functional reconstruction. Energy Convers Manag. 2021 Sep 15;244:114491.

2. McDonald D, Price MN, Goodrich J, Nawrocki EP, DeSantis TZ, Probst A, et al. An improved Greengenes taxonomy with explicit ranks for ecological and evolutionary analyses of bacteria and archaea. ISME J. 2012 Mar;6(3):610–8.

3. Beveridge T. Use of the Gram stain in microbiology. Biotech Histochem. 2001 Jan;76(3):111–8.

4. Jiajia L, Shinghung M, Jiacheng Z, Jialing W, Dilin X, Shengquan H, et al. Assessment of Neuronal Viability Using Fluorescein Diacetate-Propidium Iodide Double Staining in Cerebellar Granule Neuron Culture. J Vis Exp. 2017 May 10;(123):55442.

5. Salvador AF, Cavaleiro AJ, Paulo AMS, Silva SA, Guedes AP, Pereira MA, et al. Inhibition Studies with 2-Bromoethanesulfonate Reveal a Novel Syntrophic Relationship in Anaerobic Oleate Degradation. Appl Environ Microbiol. 2019 Jan 15;85(2):e01733-18.

6. Diener C, Gibbons SM, Resendis-Antonio O. MICOM: metagenome-scale modeling to infer metabolic interactions in the gut microbiota. mSystems. 2020 Jan 21;5(1):10–1128.

7. Parks DH, Imelfort M, Skennerton CT, Hugenholtz P, Tyson GW. CheckM: assessing the quality of microbial genomes recovered from isolates, single cells, and metagenomes. Genome Res. 2015 Jan 7;25(7):1043–55.

8. Gray N, Plumb RS, Wilson ID, Nicholson JK. A validated UPLC-MS/MS assay for the quantification of amino acids and biogenic amines in rat urine. J Chromatogr B. 2019 Feb 1;1106–1107:50–7.

9. Joseph TA, Chlenski P, Litman A, Korem T, Pe’er I. Accurate and robust inference of microbial growth dynamics from metagenomic sequencing reveals personalized growth rates. Genome Res. 2022 Mar 1;32(3):558–68.

10. Olm MR, Crits-Christoph A, Bouma-Gregson K, Firek BA, Morowitz MJ, Banfield JF. inStrain profiles population microdiversity from metagenomic data and sensitively detects shared microbial strains. Nat Biotechnol. 2021 Jun;39(6):727–36.

11. Hassa J, Wibberg D, Maus I, Pühler A, Schlüter A. Genome Analyses and Genome-Centered Metatranscriptomics of Methanothermobacter wolfeii Strain SIV6, Isolated from a Thermophilic Production-Scale Biogas Fermenter. Microorganisms. 2020 Jan;8(1):13.

12. De Bernardini N, Basile A, Zampieri G, Kovalovszki A, De Diego Diaz B, Offer E, et al. Integrating metagenomic binning with flux balance analysis to unravel syntrophies in anaerobic CO_2_ methanation. Microbiome. 2022 Aug 3;10(1):117.

13. Hori, T., Sasaki, D., Haruta, S., Shigematsu, T., Ueno, Y., Ishii, M., et al. Detection of active, potentially acetate-oxidizing syntrophs in an anaerobic digester by flux measurement and formyltetrahydrofolate synthetase (FTHFS) expression profiling. Microbiology. 2011 Jul 1;157(7):1980–9.
